# Metatranscriptomic Analysis Uncovers RNA Virus Diversity in Ticks From the China–Russia–North Korea Border Region

**DOI:** 10.1155/tbed/7807512

**Published:** 2025-10-12

**Authors:** Zhe Liu, Shengwei Ji, Jinqi Wang, Yuan Li, Eloiza May Galon, Shanshan Wang, Jixu Li, Xu Gao, Longzheng Yu, Yang Wang, Jianchen Song, Qichao Cui, Chenghui Li, Zhiqiang Xu, Shujiang Xue

**Affiliations:** ^1^College of Agriculture, Yanbian University, Yanji, Jilin, China; ^2^College of Veterinary Medicine and Biomedical Sciences, Cavite State University, Indang, Cavite, Philippines; ^3^Department of Vector Control, Yanbian Prefecture Center for Disease Control and Prevention, Yanji, Jilin, China

**Keywords:** China–Russia–North Korea border region, metatranscriptomic analysis, tick

## Abstract

Ticks serve as critical viral vectors, and border regions, acting as convergence zones of complex ecosystems, provide diverse habitats for ticks and their hosts, thereby underscoring the need to investigate the tick-borne virome composition in such areas. In this study, metatranscriptomic analysis of five tick species, namely *Haemaphysalis longicornis*, *Haemaphysalis concinna*, *Haemaphysalis japonica*, *Ixodes persulactus*, and *Dermacentor silvarum*, collected from the China–Russia–North Korea border region identified 10 viral families and 22 viral species. Among these, five were confirmed human pathogens, while nine exhibited potential zoonotic risks. Moreover, significant variations in virome composition across sampling sites revealed associations between tick-borne viruses and ecological-geographical factors. These findings highlight the diversity and spatiotemporal distribution patterns of tick-borne viruses in the region, offering critical insights for safeguarding border biosecurity and public health.

## 1. Introduction

Ticks rank as the second most significant natural vectors of arboviral diseases, second only to mosquitoes, with over 800 species identified globally. These arthropods are capable of transmitting more than 200 pathogens, including over 160 tick-borne viruses, posing substantial threats to public health [1]. Approximately 30% of tick-borne viruses are associated with severe human diseases, exemplified by Xue-Cheng virus (XCV) [2], Songling virus (SGLV) [3], Beiji nairovirus (BJNV) [4, 5], tick-borne encephalitis virus (TBEV) [6–9], Wetland virus (WELV) [10], Crimean-Congo hemorrhagic fever virus (CCHFV) [11, 12], and Alongshan virus (ALSV) [13–15]. In recent years, intensified research on tick-borne pathogens has led to the discovery of numerous novel agents, coinciding with rising incidence rates of tick-borne diseases across regions. This trend reinforces the existence of a vast reservoir of undiscovered, potentially pathogenic tick-borne agents awaiting characterization [16–19].

Next-generation sequencing (NGS) has been extensively employed for monitoring pathogens in humans, animals, and plants [1]. In China, metatranscriptomic analysis of serum samples from febrile patients in 2025 first identified XCV, a novel tick-borne orthonairovirus belonging to the *Orthonairovirus* genus within the Nairoviridae family, with subsequent detection in *Haemaphysalis concinna* and *H. japonica* [2]. SGLV, initially discovered in a patient in 2021 [20], has since been documented in Xinjiang gerbils [21] and diverse tick species across multiple Chinese regions [22–24]. Similarly, WELV, a novel pathogen linked to multiorgan dysfunction, was first isolated via NGS analysis and is predominantly distributed in Inner Mongolia, Jilin, Liaoning, and Heilongjiang provinces [25]. Metagenomic sequencing has emerged as a pivotal tool for dynamically surveilling known and emerging pathogens, elucidating viral evolution patterns, and providing critical insights into transmission mechanisms and public health risk assessments.

The China–Russia–North Korea border region, characterized by rich biodiversity and frequent cross-border trade activities [26], is highly sensitive to climate change, with an annual temperature increase rate exceeding the national average. This climatic shift has prolonged tick activity periods, significantly elevating the risk of emerging infectious diseases [27]. To date, multiple zoonotic viruses have been identified in this area, including WELV [10], Tamdy orthonairovirus (TDOV) [20], TBEV [24], severe fever with thrombocytopenia syndrome virus (SFTSV) [28], Nairobi sheep disease virus (NSDV) [28], Onega tick phlebovirus (OTPV) [29], and ALSV [22]. These findings highlight the need to strengthen surveillance of tick-borne diseases in this ecologically vulnerable zone. In this study, we conducted molecular epidemiological investigations on ticks collected from the region through metatranscriptomic analysis, aiming to delineate the geographical distribution of tick species and their associated viral prevalence. Our objectives were to delineate tick species distribution and determine the prevalence of tick-borne viruses, thereby providing critical data for assessing cross-border transmission risks and formulating targeted biosecurity measures to mitigate public health threats.

## 2. Materials and Methods

### 2.1. Sample Collection and Identification

**Figure 1:**
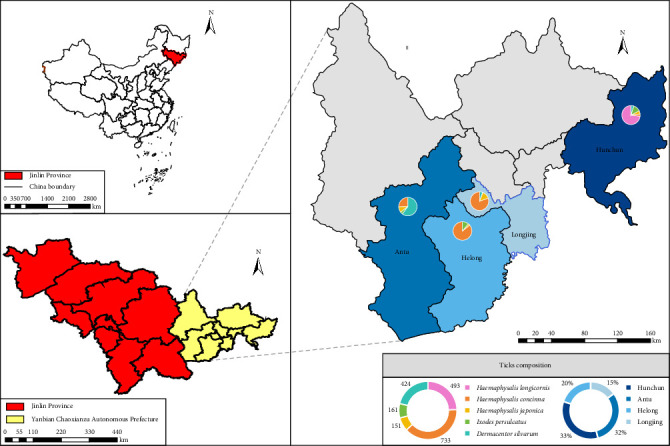
Distribution of tick collection sites along the China–Russia–North Korea border area. The map was made using ArcMap 10.8 software, and the data was obtained from https://datav.aliyun.com/portal and http://www.resdc.cn/.

From April to July 2023 and 2024, ticks were collected from four regions (Antu County, Hunchun City, Helong City, and Longjing City) within the China–Russia–North Korea border region using the woolen flannel cloth dragging method (Figure 1). Following field collection, specimens were temporarily stored on dry ice during transportation and subsequently transferred to −80°C ultra-low temperature freezers for long-term preservation in laboratory facilities. Collected tick specimens underwent preliminary morphological identification via stereomicroscopy using taxonomic identification keys [30], followed by molecular confirmation through sequencing of the mitochondrial cytochrome c oxidase subunit I (COI) gene [31, 32].

### 2.2. RNA Library Construction and Sequencing

The collected tick specimens were categorized into 287 pools based on collection sites, species, and sex (Supporting Information 1). Specimens underwent sequential decontamination through immersion in 75% ethanol followed by three washes with phosphate-buffered saline (PBS, pH 7.4) [33]. Each pool was homogenized in 500 μL Dulbecco's Modified Eagle's Medium (DMEM) supplemented with two 3 mm zirconium beads using the high-throughput tissue grinder (Jingxin, China) at 60 Hz for 10 min. Homogenates were centrifuged at 13,400 × *g* for 15 min at 4°C to pellet cellular debris. Supernatants were sequentially filtered through 0.45 and 0.22 μm Millex membrane filters (Millipore, USA) to eliminate residual particulates. Filtered lysates were aliquoted into two fractions: one for RNA library construction and the other for virome characterization.

The 287 filtrate samples were categorized into four libraries based on sampling locations. Free nucleic acids in the filtrate were enzymatically digested prior to total RNA extraction using the QIAamp Viral RNA Mini Kit (Qiagen, Germany). Total RNA was subsequently fragmented, and double-stranded cDNA was synthesized via random hexamer priming and dNTP incorporation, followed by adapter ligation, single-primer PCR amplification, and purification according to the manufacturer's protocol. Metatranscriptomic analysis was conducted by Shanghai Personalbio Technology Co., Ltd. utilizing the Illumina NovaSeq 6000 platform.

### 2.3. Bioinformatics Analysis

Raw sequencing data from each library were processed using fastp (v0.20.0) [34] to remove low-quality sequences, including short reads and adapter-contaminated fragments. Cleaned reads were then aligned to the host genome using minimap2 (v2.24) [35] to eliminate host-derived contamination. De novo assembly of the remaining high-quality reads was performed with MEGAHIT (v1.2.9) [36] under the meta-sensitive preset parameters, retaining contigs ≥300 bp in length. To recover fragmented sequences, unmapped reads from each sample were realigned to their corresponding contigs using minimap2 and subjected to iterative reassembly. Taxonomic annotation of contigs was conducted via Kaiju [37] and DIAMOND BLASTp [38] against the NCBI nr and RefSeq-Viral databases, with stringent thresholds set at a maximum of five mismatches and an *E*-value cutoff of 1 × 10^−5^ to minimize false-positive identifications [37, 39].

### 2.4. Viral Screening

To validate the Illumina sequencing results, primers were designed using Primer 6 (Premier Biosoft International, USA; primer sequences listed in Supporting Information 2). Viral RNA was extracted using the QIAamp Viral RNA Mini Kit (Qiagen, Germany), followed by cDNA synthesis with FastKing gDNA Dispelling RT SuperMix (Tiangen, China). PCR amplification was performed using PCR Master Mix (Tiangen, China), and all amplicons were resolved on 1.0% agarose gels. Positive amplification products were purified using the TIANgel Midi Purification Kit (Tiangen, China) and subsequently subjected to Sanger et al. [40] sequencing for verification.

### 2.5. Phylogenetic Analysis

To confirm the phylogenetic relationships of the viral strains identified in this study, complete and partial nucleotide sequences were subjected to BLASTn analysis against the NCBI database to retrieve homologous reference sequences. Multiple sequence alignment was performed using MAFFT v7.526 [41], followed by maximum-likelihood phylogenetic tree construction with IQ-TREE v2.4.0 [42] under 1000 ultrafast bootstrap replicates. Final tree visualization and annotation were implemented in FigTree v1.4.4 (http://tree.bio.ed.ac.uk/software/figtree/), with midpoint rooting applied in the absence of a predefined outgroup and nodes ordered by descending support values.

### 2.6. Statistical Analysis

The viral infection rates of ticks were calculated using PooledInfRate software, version 4.0 (a Microsoft Office Excel Add-In designed by Brad J. Biggerstaff to compute prevalence estimates from pooled samples, Centers for Disease Control and Prevention, Fort Collins, CO, USA, 2024). The prevalence of viruses in ticks was presented as the maximum likelihood estimate (MLE) per 100 ticks with a 95% confidence interval (95% CI). The data were analyzed using SPSS version 21.0.

## 3. Results

### 3.1. Tick Collection and Identification

A total of 1962 ticks were collected from the China–Russia–North Korea border region and classified into five species: *H. longicornis* (*n* = 493), *H. concinna* (*n* = 733), *H. japonica* (*n* = 151), *I. persulcatus* (*n* = 161), and *D. silvarum* (*n* = 424) (Figure 1 and Table 1). Sampling sites included Helong City (*n* = 416), Hunchun City (*n* = 676), Antu County (*n* = 578), and Longjing City (*n* = 292) (Supporting Information 1). Analysis revealed that *H. concinna*, *H. japonica*, and *I. persulcatus* were distributed across all four sampling sites, whereas *H. longicornis* was exclusively detected in Hunchun City.

### 3.2. Detection and Statistical Analysis of RNA Viruses

Four RNA virome libraries were constructed and sequenced, generating 90 GB of raw data. Following quality filtering, 1,727,344 clean reads were retained. De novo assembly yielded 4526 viral contigs, with viral reads representing 0.06%–0.75% of non-rRNA reads per library. Viral sequences were annotated into nine viral families and one unclassified family, encompassing 22 viral species (Table 2), based on BLASTn analysis (E-value ≤1 × 10^−5^). Notably, five RNA virus families with zoonotic potential were identified: Flaviviridae, Nairoviridae, Phenuiviridae, Chuviridae, and Rhabdoviridae. Specific viruses included XCV, SGLV, Dabieshan tick virus (DBTV), Mukawa virus (MKWV), BJNV, Manly virus (MLV), Sara tick phlebovirus (STPV), Hunchun nairovirus (HCNV), Ji'an nairovirus (JANV), Yanbian Rhabd tick virus 1 (YBRV1), Yanbian Rhabd tick virus 4 (YBRV4), Tahe rhabdovirus 1 (THRV1), Lesnoe mivirus (LMV), and Yanggou tick virus (YGTV). Analysis of viral read abundance revealed that the genus *Orthonairovirus* within the family Nairoviridae exhibited the highest abundance, primarily represented by SGLV. First identified in 2021, SGLV is transmissible via ticks to diverse animals and humans, inducing febrile illnesses and headaches, thereby posing a significant public health threat [20]. The second most abundant reads were attributed to YGTV, a member of the Jingmenvirus group within the family Flaviviridae. To validate the virome results, we performed PCR assays for dual verification of the aforementioned viruses and systematically analyzed their geographic and tick-species distribution patterns (primer sequences are detailed in Supporting Information 2). All amplified sequences have been deposited in the NCBI database (GenBank accession numbers are available in Table 3 and Supporting Information 3).

Viral species and prevalence demonstrated marked geographic heterogeneity and tick-species specificity across four sampled regions. Hunchun exhibited the highest viral diversity with 14 species detected, while Helong showed the lowest, containing only three species (Supporting Information 4). Notably, only SGLV and LMV were detected in all four regions. LMV reached its highest prevalence in Helong (3.40%, 13/58), whereas SGLV peaked in Longjing (5.80%, 14/40). Additionally, a novel human-associated virus, XCV, was identified in Hunchun with a prevalence of 0.30% (2/107). Other viruses with zoonotic potential detected in this region included DBTV (5.77%, 31/107), HCNV (0.60%, 4/107), JANV (0.29%, 2/107), and STPV (0.15%, 1/107), with DBTV being the most prevalent. Antu harbored the highest variety of potentially human-infective viruses, encompassing SGLV (1.23%, 7/82), STPV (0.35%, 2/82), YGTV (0.35%, 2/82), THRV1 (0.35%, 2/82), YBRV4 (0.35%, 2/82), YBRV1 (0.17%, 1/82), and MLV (0.35%, 2/82), where SGLV showed the highest prevalence and YBRV1 the lowest. Helong and Longjing each yielded one human-associated virus: BJNV (0.24%, 1/58) and MKWV (0.34%, 1/40), respectively (Table 4; Supporting Information 5).

Several viruses demonstrated distinct geographic restrictions: BJNV was exclusively detected in Helong; DBTV, XCV, HCNV, JANV, CLTV3, HNTV, Hepelivirales sp., and ISAV1 were found only in Hunchun; YGTV, THRV1, YBRV1, YBRV4, and MLV were unique to Antu; while MKWV and NXLV were solely present in Longjing.

Analysis at the tick species level revealed that *I. persulcatus* carried the highest viral diversity, with eight viruses detected. In Hunchun, *I. persulcatus* harbored six viruses: SGLV (1.19%, 1/15), HCNV (5.00%, 4/15), STPV (1.23%, 1/15), JANV (1.14%, 1/15), JLPV1 (1.14%, 1/15), and ISAV1 (1.14%, 1/15), with HCNV exhibiting the highest prevalence. *I. persulcatus* from Antu carried three human-associated viruses—SGLV, YBRV1, and STPV—among which STPV showed the highest prevalence (9.11%, 2/9). Only BJNV was detected in *I. persulcatus* from Helong, at a prevalence of 2.19% (1/11), whereas no viruses were identified in *I. persulcatus* from Longjing. Among all viruses, the highest prevalence was observed for LMV in *H. japonica* from Helong (43.82%, 4/6), and the lowest for both MLV and THRV1 in *D. silvarum* from Antu (0.27%, 1/44 each) (Supporting Information 6).

### 3.3. RNA Viruses Diversity and Evolution

#### 3.3.1. Phenuiviridae

**Figure 2:**
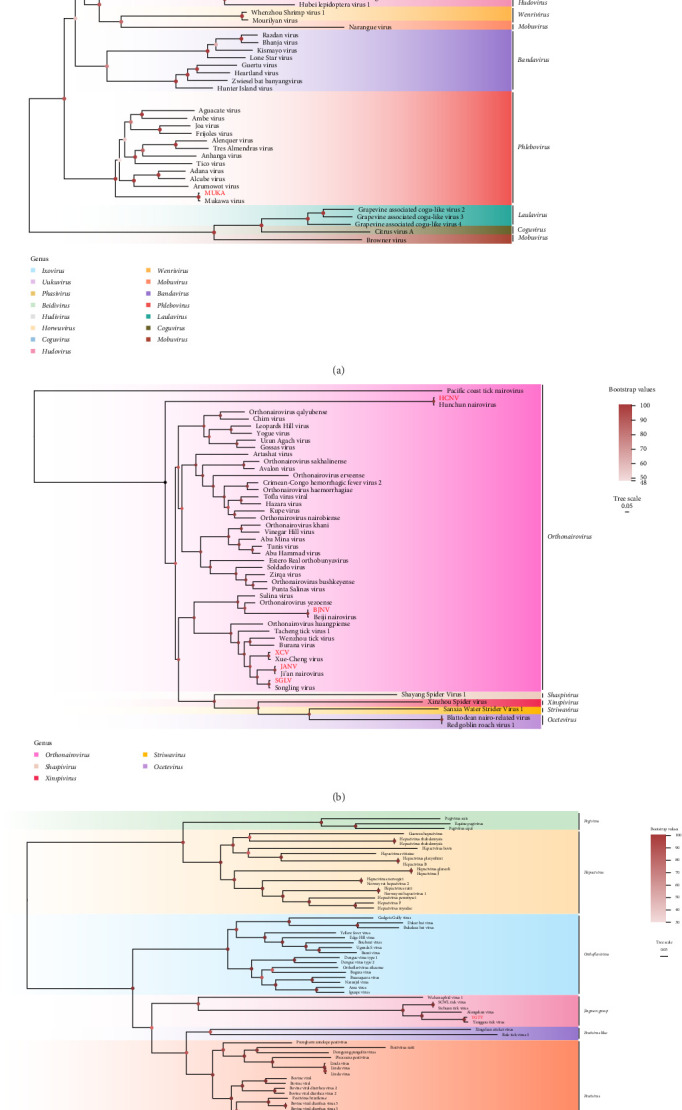
Phylogenetic analysis of Phenuiviridae (A), Nairoviridae (B), and Flaviviridae (C). All the viruses obtained in this study were highlighted in red. BJNV, Beiji nairovirus; DBTV, Dabieshan tick virus; HCNV, Hunchun nairovirus; MKWV, Mukawa phlebovirus; SGLV, Songling virus; STPV, Sara tick phlebovirus; XCV, Xue-Cheng virus; YGTV, Yanggou tick virus. The accession numbers of the viral sequences are shown in Supporting Information 3.

The family Phenuiviridae, classified within the order Bunyavirales, comprises single-stranded negative-sense RNA viruses and encompasses several genera, including *Phlebovirus*, *Bandavirus*, *Uukuvirus*, *Ixovirus*, and *Hudivirus* [43–45]. In this study, three Phenuiviridae were identified: DBTV (520 bp), MKWV (895 bp), and STPV (443 bp). Among these, DBTV, phylogenetically assigned to the genus *Uukuvirus*, was exclusively detected in *H. longicornis* from the Hunchun region, exhibiting nucleotide sequence similarities of 98.80%–99.31% with other strains isolated from the same tick species. Similarly, MKWV was uniquely identified in *D. silvarum* from Longjing, sharing nucleotide identities of 98.18%–98.72% with other strains. In contrast, STPV, classified under the *Ixovirus* genus, was detected in *I. persulcatus* across both the Hunchun and Antu regions, showing 92.75%–99.78% nucleotide identity with other strains (Figure 2A and Table 2).

#### 3.3.2. Nairoviridae

The genus *Orthonairovirus* within the family Nairoviridae comprises at least seven viral clusters encompassing 15 species, many of which pose significant threats to human and animal health [5, 46, 47]. These viruses are primarily transmitted by ticks and possess segmented single-stranded RNA genomes. In this study, five *Orthonairovirus* species were identified in tick specimens: SGLV (500 bp), BJNV (498 bp), HCNV (457 bp), JANV (1375 bp), and XCV (766 bp). SGLV was detected in *H. concinna* from Helong, Longjing, and Antu, as well as in *I. persulcatus* from Antu and *H. longicornis* from Hunchun, exhibiting nucleotide identities of 96.92%–99.45% with other strains. HCNV and BJNV were exclusively identified in *I. persulcatus* from Hunchun and Helong, respectively, while JANV was detected in both *H. concinna* and *I. persulcatus* from Hunchun, with nucleotide identities of 98.99%–99.82%, 99.18%, and 99.18%–99.52%, respectively. Additionally, XCV, a recently discovered zoonotic virus, was identified in *H. japonica* and *H. concinna* from Hunchun, sharing 96.88%–99.03% nucleotide identity with othoer strains isolated from *H. concinna* (Figure 2B and Table 2).

#### 3.3.3. Flaviviridae

The family Flaviviridae comprises single-stranded positive-sense RNA viruses and encompasses four primary genera along with unclassified groups, including *Orthoflavivirus*, *Hepacivirus*, *Pestivirus*, *Pestivirus*-like, *Pegivirus*, and the Jingmenvirus group [48]. In recent years, *Flaviviruses* have been increasingly implicated in global outbreaks, posing significant threats to both human and animal health [13]. In this study, a segmented *Flavivirus*, YGTV (300 bp), was identified in *D. silvarum* from the Antu region. YGTV exhibited nucleotide sequence similarities of 96.72%–99.67% with other YGTV strains (Figure 2C and Table 2).

#### 3.3.4. Chuviridae

**Figure 3:**
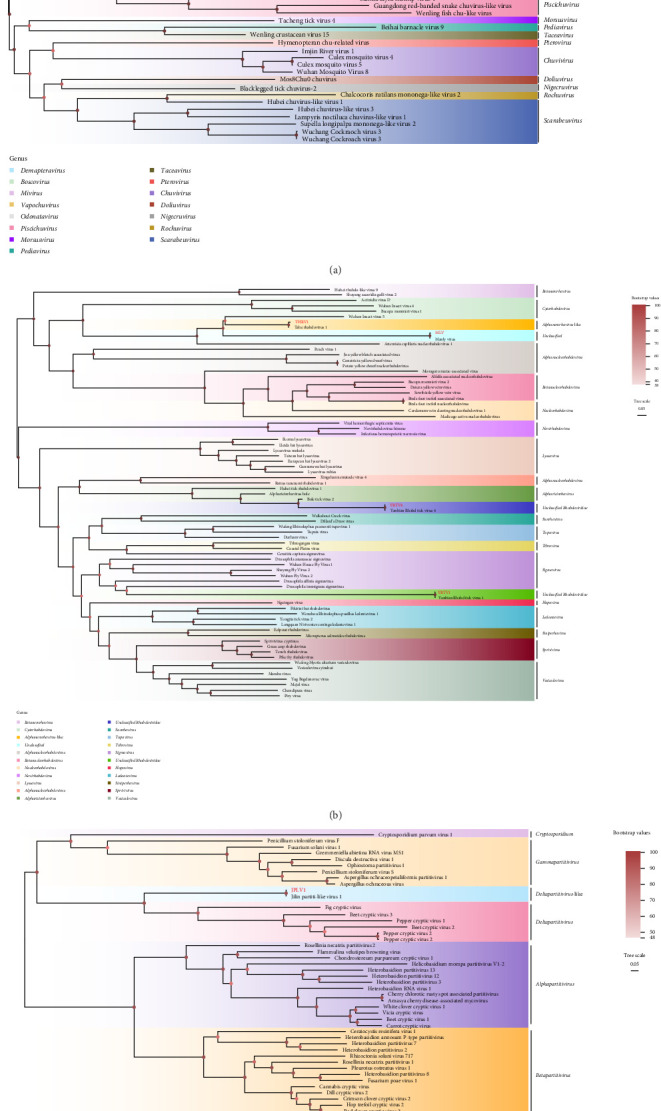
Phylogenetic analysis of Chuviridae (A), Rhabdoviridae (B), and Partitiviridae (C). All the viruses obtained in this study were highlighted in red. JLPV1, Jilin partiti-like virus 1; LMV, Lesnoe mivirus; MLV, Manly virus; THRV1, Tahe rhabdovirus 1; YBRV1, Yanbian Rhabd tick virus 1; YBRV4, Yanbian Rhabd tick virus 4. The accession numbers of the viral sequences are shown in Supporting Information 3.

The virus was initially discovered in arthropods from Xinjiang, China, and includes members such as *Mivirus*, *Demapteravirus*, *Boscovirus*, *Morsusvirus*, and *Scarapeuvirus* [49]. In this study, LMV (1281 bp), classified under the genus *Mivirus* within the family Chuviridae, was detected in *H. concinna*, *H. japonica*, and *D. silvarum* from Longjing, and presented 98.75%–100% nucleotide identity with other strains (Figure 3A and Table 2).

#### 3.3.5. Rhabdoviridae

Members of the family Rhabdoviridae exhibit an exceptionally broad host range, infecting mammals, arthropods, fish, and plants [50–52]. These viruses possess single-stranded negative-sense RNA genomes characterized by diverse structural features [33]. In this study, three *Rhabdoviruses* were detected in ticks from the Antu region: THRV1 (239 bp), *Alphanemrhavirus*-like group, and two unclassified *Rhabdovirids*, YBRV4 (579 bp), YBRV1 (822 bp) and MLV (764 bp). The nucleotide identities of these strains were 99.37%–99.61%, 99.04%–99.54%, 99.70%–99.74%, and 99.68%, respectively (Figure 3B and Table 2).

#### 3.3.6. Partitiviridae

The family Partitiviridae, encompassing double-stranded RNA viruses, includes genera such as *Deltapartitivirus*-like, *Cryspovirus*, *Alphapartitivirus*, *Betapartitivirus*, *Deltapartitivirus*, and *Gammapartitivirus* [53]. In this study, Jilin partiti-like virus 1 (JLPV1) (345 bp), *Deltapartitivirus*-like, was detected in *I. persulcatus* from both Hunchun and Antu. JLPV1 shared nucleotide identities of 99.68% with other strains (Figure 3C and Table 2).

#### 3.3.7. Tombusviridae

**Figure 4:**
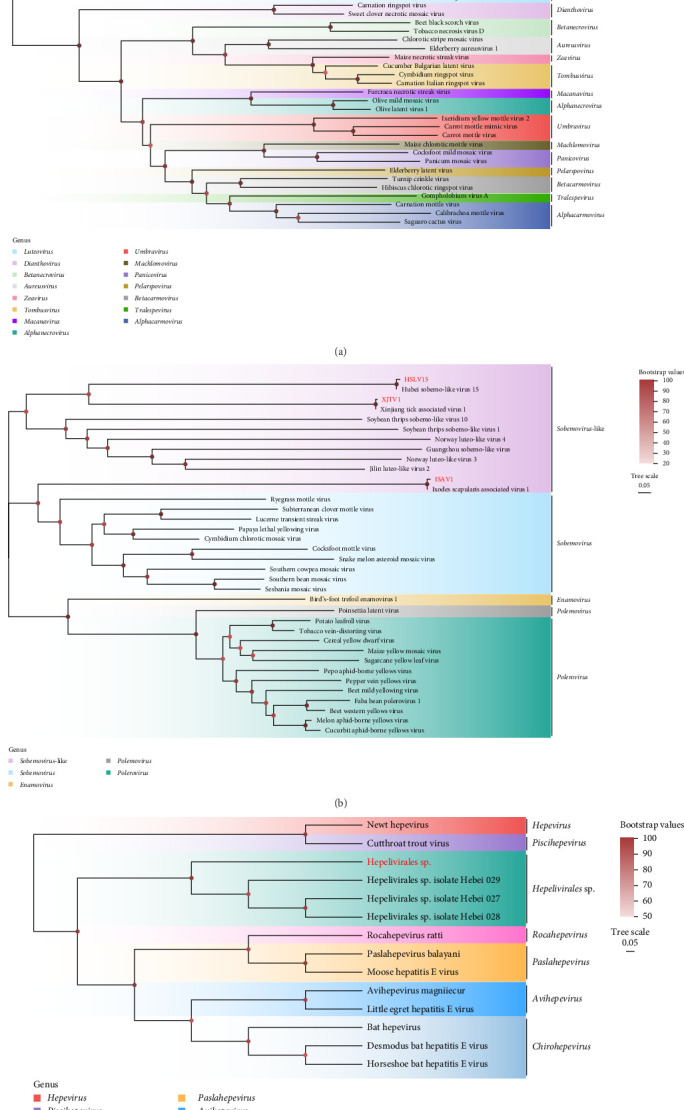
Phylogenetic analysis of Tombusviridae (A), Solemoviridae (B), and Hepeviridae (C). All the viruses obtained in this study were highlighted in red. CLTV3, Cheeloo tick virus 3; HSLV15, Hubei sobemo-like virus 15; ISAV1, Ixodes scapularis associated virus 1; NXLV, Ningxia luteovirus; XTAV1, Xinjiang tick associated virus 1. The accession numbers of the viral sequences are shown in Supporting Information 3.

The family Tombusviridae, known to primarily infect plants and induce symptoms such as leaf mottling, chlorosis, and localized necrosis, has been reported across multiple European countries and North America [54]. In this study, two viruses belonging to *Luteovirus* within this family―CLTV3 (1436 bp) and NXLV (1439 bp)―were identified in ticks. NXLV was detected in *H. japonica* from Longjing, exhibiting nucleotide sequence identities of 97.78%–98.09% with other strains isolated from *H. japonica*. CLTV3, identified in *H. longicornis* from Hunchun, shared nucleotide identities of 96.19%–99.31% with previously discovered strains detected in *H. longicornis* (Figure 4A and Table 2).

#### 3.3.8. Solemoviridae

Viruses within the family Solemoviridae are predominantly distributed in tropical and subtropical regions and rank among the most devastating plant pathogens globally [55, 56]. Researchers identified 22 putative novel *Solemoviridae* in 2025 [57]. In this study, three viruses belonging to the Sobemo-like family―Xinjiang tick associated virus 1 (XTAV1) (754 bp), Hubei sobemo-like virus 15 (HSLV15) (534 bp), and ISAV1 (324 bp)―were detected. XTAV1 was exclusively identified in *D. silvarum* from the Antu region, exhibiting nucleotide sequence identities of 98.59%–99.42% with other strains isolated from the same tick species. HSLV15 was detected in *H. concinna*, *H. japonica*, and *D. silvarum* ticks from Longjing, as well as in *H. japonica* and *H. longicornis* from Hunchun, with shared nucleotide identities ranging from 97.07%–98.70% with other strains. ISAV1, detected in *I. persulcatus* from Hunchun, shared 98.79% nucleotide identity with other strains (Figure 4B and Table 2).

#### 3.3.9. Hepeviridae

The family Hepeviridae comprises multiple genera, including *Hepevirus*, *Hepelivirales* sp., *Piscihepevirus*, *Avihepevirus*, *Chirohepevirus*, and *Orthohepevirus* [58]. In this study, contigs classified under *Hepelivirales* sp. (879 bp) were identified in *H. longicornis* from Hunchun. *Hepelivirales* sp. shared 96.14%–98.73% nucleotide identity with previously discovered strains detected in *H. longicornis*.

## 4. Discussion

Recent advances in metatranscriptomic analysis have facilitated the discovery of an increasing number of novel tick-borne viruses, significantly enriching the diversity of tick-borne viral species [44, 59–62]. In this study, metatranscriptomic analysis of ticks from the China–Russia–North Korea border region provided the first comprehensive characterization of viral composition in this ecologically sensitive area. In the five tick species analyzed, we identified 22 viral species spanning 10 families: Flaviviridae, Nairoviridae, Phenuiviridae, Chuviridae, Rhabdoviridae, Tombusviridae, Partitiviridae, Solemoviridae, Hepeviridae, and unclassified. Among these, five species are confirmed human pathogens, while nine exhibit potential zoonotic risks.

Previous metatranscriptomic analysis of *I. persulcatus* in the China–Russia–North Korea border region identified YGTV, STPV, MKWV, and BJNV, all of which were reconfirmed in this study [33]. Notably, XCV, a novel tick-borne *Orthonairovirus* initially discovered in a febrile patient, in *H. concinna*, and *H. japonica* from Mudanjiang City, Heilongjiang Province in 2025 [2], was also detected in *H. japonica* and *H. concinna* from Hunchun. This finding corroborates prior reports and suggests potential geographical expansion beyond the originally identified Mudanjiang focus, indicating broader regional dissemination. DBTV, currently widespread in Japan [63] and Chinese provinces such as Shandong and Hubei [18, 64, 65], has been classified within the *Orthohantavirus dabieshanense* species and is closely related to Hantaan virus (HTNV). It has been stated that DBTV can cause hemorrhagic fever with renal syndrome (HFRS) in humans [66]. Additionally, seropositivity for DBTV antibodies was detected in Shandong sheep sera [67]. Han et al. [66] postulated a coastal distribution pattern for DBTV, with prevalence correlating strongly with habitats suitable for its primary vector *H. longicornis*, and migratory bird activity as a key dispersal drivers [68]. Bai et al. [28] reported high DBTV prevalence in Liaoning border ticks, consistent with our findings of elevated positivity rates in *H. longicornis* from Hunchun. The broad distribution of DBTV underscores the urgent need to elucidate its pathogenesis and genetic traits while addressing risks of cross-border spread via wildlife migration or trade activities. In 2017, Li et al. [69] investigated SFTSV in ticks from the Yanbian region, reporting a minimum infection rate of 1.81%. Notably, no SFTSV-positive ticks have been reported in subsequent studies conducted post-2017 in this region, a finding corroborated in the current study. SGLV was detected across all four sampling sites, with *H. concinna* identified as the predominant tick species. Higher SGLV positivity rates were observed in Helong and Longjing, whereas lower prevalence was recorded in neighboring Hunchun and Antu, likely attributable to localized ecological heterogeneity among the regions. In 2023, Li et al. [23] identified a novel *Orthonairovirus*, Antu virus, in *D. silvarum* from the China–North Korea border region of Jilin Province. Phylogenetic analysis revealed its close relationship to SGLV, suggesting potential zoonotic capacity. However, Antu virus was not detected in *D. silvarum* from Antu in the current study, possibly due to temporal variations in tick sampling or degradation of low-titer viral RNA under noncontrolled conditions [70]. BJNV, classified under the genus *Orthonairovirus* within the family Nairoviridae, lacks the medium (M) genomic segment encoding viral glycoproteins [10]. First identified in *I. persulcatus* in 2014 [10, 22, 24], BJNV was later reported in China–North Korea border regions by Wang et al. [29] in 2017. To date, BJNV has only been documented in northeastern China. Our findings confirm its persistence in *I. persulcatus* from Helong in 2025, indicating the establishment of stable natural foci in this region. The maintenance of BJNV may involve vertical transmission in ticks or sustained multihost cycles among wildlife reservoirs, underscoring the necessity for continuous surveillance of tick-borne pathogens.

Furthermore, molecular epidemiological investigations of tick-borne viruses across four sampling sites in the China–Russia–North Korea border region revealed host-specific viral distributions. For instance, XTAV1 and STPV were exclusively detected in *D. silvarum*, while DBTV was restricted to *H. longicornis*, consistent with findings by Xu et al. [65]. This phenomenon may be attributed to the virus's reliance on amplifying hosts that have established a closed-loop transmission cycle exclusively with *H. longicornis*, while other tick species exhibit inefficient viral acquisition or transmission due to low host contact frequency. Additionally, transovarial transmission of DBTV in *H. longicornis* [64] could further explain its host-restricted distribution [71]. Notably, viral prevalence exhibited marked geographic heterogeneity: tick pools from Hunchun, Helong, Longjing, and Antu showed positivity rates of 11.33% (53/107), 6.13% (21/58), 14.45% (27/40), and 4.49% (23/82), respectively. Rhabdoviridae and Flaviviridae were uniquely identified in Antu, whereas Hepeviridae was confined to Hunchun. These patterns likely arise from multifactorial drivers, including animal migration, climatic variability, anthropogenic activities, and habitat modifications [72].

This study has several limitations. While we confirmed the presence of several human disease-associated viruses in the region, several previously documented tick-borne viruses—including Antu virus [23], Tacheng tick virus-1 (TcTV-1) [22], ALSV [29], and NSDV [28]—were undetected. This discrepancy may be attributed to multifactorial influences, including potential mismatches between the spatiotemporal sampling coverage and viral activity cycles, as well as genetic variations arising from viral mutation or evolutionary divergence. Thus, expanding the geographical scope of metatranscriptomic analyses and optimizing cross-seasonal sampling strategies will facilitate deeper elucidation of viral transmission dynamics. Furthermore, the absence of systematic environmental parameters (e.g., humidity and forest coverage) and human activity data from sampling sites constrains deeper exploration of viral dissemination patterns and evolutionary trajectories, which will constitute a priority for future investigations.

## 5. Conclusions

In conclusion, this study conducted metatranscriptomic analyses and prevalence surveys of tick-borne viruses in the China–Russia–North Korea border region, providing the first systematic assessment of the tick-borne virome in this area and revealing their potential public health risks. These findings not only enhance our capacity to predict the spatiotemporal distribution and transmission dynamics of these viruses but also lay a critical data foundation for cross-border joint prevention and control strategies, as well as risk assessment and early warning of emerging infectious diseases.

## Figures and Tables

**Table 1:** Distribution of tick species across the four sampling locations.

Collection site	Number of individuals/pools	*Haemaphysalis longicornis*	*Haemaphysalis concinna*	*Haemaphysalis japonica*	*Ixodes persulcatus*	*Dermacentor silvarum*
Helong	416/58	0/0	358/41	12/6	46/11	0/0
Hunchun	676/107	493/59	27/11	37/11	85/15	34/11
Antu	578/82	0/0	114/17	62/12	28/9	374/44
Longjing	292/40	0/0	234/27	40/7	2/1	16/5
Total	1962/287	493/59	733/96	151/36	161/36	424/60

**Table 2:** Viruses identified in the present study.

Classification	Virus (abbreviation)	Contig length (bp)	Closest relative (accession number)	Closest relative (% nt identity)	Number of unique contigs
Flaviviridae					
* Jingmenvirus*	Yanggou tick virus (YGTV)	2439–2748	Yanggou tick virus isolate YGTV_YBQG1718A	96.72%–99.67%	2
Nairoviridae					
* Orthonairovirus*	Songling virus (SGLV)	777–12,001	Songling virus strain NE-TH2 segment L	96.92%–99.45%	17
* Orthonairovirus*	Beiji nairovirus (BJNV)	525	Beiji nairovirus isolate BJNV_YBQG1717	99.18%	1
* Orthonairovirus*	Hunchun nairovirus (HCNV)	406–1635	Hunchun nairovirus isolate NE-FCH1	98.99%–99.82%	9
* Orthonairovirus*	Ji'an nairovirus (JANV)	339–1113	Ji'an nairovirus isolate NE-DGZ3	99.18%–99.52%	4
* Orthonairovirus*	Xue-Cheng virus (XCV)	639–739	Xue-Cheng virus isolate TIGMIC 1	96.88%–99.03%	2
Phenuiviridae					
* Uukuvirus*	Dabieshan tick virus (DBTV)	426–6518	Dabieshan Tick Virus isolate TIGMIC_48	98.80%–99.31%	5
* Ixovirus*	Sara tick phlebovirus (STPV)	423–924	Sara tick phlebovirus isolate STPV_BSQG1730	92.75%–99.78%	3
* Phlebovirus*	Mukawa phlebovirus (MKWV)	606–860	Mukawa virus strain NE-FZ2 segment M	98.18%–98.72%	3
Chuviridae					
* Mivirus*	Lesnoe mivirus (LMV)	591–5172	Lesnoe mivirus isolate TIGMIC_14	98.75%–100%	5
Rhabdoviridae					
* Alphanemrhavirus*-like	Tahe rhabdovirus 1 (THRV1)	732–873	Tahe rhabdovirus 1 strain NE-DH1	99.37%–99.61%	3
Unclassified Rhabdoviridae	Yanbian Rhabd tick virus 4 (YBRV4)	627–655	Yanbian Rhabd tick virus 4 isolate TIGMIC 5	99.04%–99.54%	3
Unclassified Rhabdoviridae	Yanbian Rhabd tick virus 1 (YBRV1)	669–1167	Yanbian Rhabd tick virus 1 isolate TIGMIC 4	99.70%–99.74%	2
Unclassified Rhabdoviridae	Manly virus (MLV)	333	Manly virus isolate HLJ-HC-3	99.68%	1
Tombusviridae					
* Luteovirus*	Cheeloo tick virus 3 (CLTV3)	717–1050	Cheeloo tick virus 3 isolate Hebei_030 segment 1	96.19%–99.31%	3
* Luteovirus*	Ningxia luteovirus (NXLV)	885–1008	Ningxia luteovirus strain China-NX143	97.78%–98.09%	2
Partitiviridae					
* Deltapartitivirus*-like	Jilin partiti-like virus 1 (JLPV1)	627	Jilin partiti-like virus 1 isolate JL/QG-2	99.68%	1
*Solemoviridae*					
Sobemo-like	Xinjiang tick associated virus 1 (XTAV1)	504–1353	Xinjiang tick associated virus 1 strain DH2	98.59%−99.42%	3
Sobemo-like	*Ixodes scapularis* associated virus 1 (ISAV1)	450	*Ixodes scapularis* associated virus 1 strain YC4	98.79%	1
Sobemo-like	Hubei sobemo-like virus 15 (HSLV15)	1059–1614	Hubei sobemo-like virus 15 strain China-NX143	97.07%–98.70%	5
Hepeviridae					
Unclassified Hepeviridae	Hepelivirales sp.	708–1896	Hepelivirales sp. isolate Hebei_027	96.14%–98.73%	4
Unclassified	Henan tick virus (HNTV)	84–1203	Henan tick virus isolate CLCM-060 segment S	94.21%–96.25%	5

**Table 3:** Accession numbers of viral sequences amplified by PCR in the present study.

Viral species	Collection sites	GenBank accession number
Lesnoe mivirus	Helong	PV641990–PV642002
	Hunchun	PV642005–PV642014
	Longjing	PV641981–PV641989
	Antu	PV642003, PV642004

Songling virus	Longjing	PV642059–PV642072
	Helong	PV642052–PV642058
	Antu	PV642073–PV642079
	Hunchun	PV642080–PV642083

Dabieshan tick virus	Hunchun	PV642021–PV642051

Xinjiang tick associated virus 1	Antu	PV641964–PV641969
	Longjing	PV641971, PV641972
	Hunchun	PV641970

Cheeloo tick virus 3	Hunchun	PV642095–PV642101

Hubei sobemo-like virus 15	Longjing	PV641976–PV641978
	Hunchun	PV641973–PV641975

Hepelivirales sp.	Hunchun	PV642088–PV642093

Hunchun nairovirus	Hunchun	PV642015–PV642018

Yanbian Rhabd tick virus 4	Antu	PV641961, PV641962

Yanbian Rhabd tick virus 1	Antu	PV641956

Sara tick phlebovirus	Antu	PV641951, PV641952
	Hunchun	PV641953

Xue-Cheng virus	Hunchun	PV642019, PV642020

Ji'an nairovirus	Hunchun	PV641979, PV641980

Yanggou tick virus	Antu	PV642084, PV642085

Manly virus	Antu	PV641954, PV641955

Jilin partiti-like virus 1	Antu	PV641959
	Hunchun	PV641960

Tahe rhabdovirus 1	Antu	PV641957, PV641958

*Ixodes scapularis* associated virus 1	Hunchun	PV641963

Beiji nairovirus	Helong	PV642094

Mukawa phlebovirus	Longjing	PV642086

Ningxia luteoviru	Longjing	PV642087

**Table 4:** PCR survey results for the viral testing of ticks collected in China–Russia–North Korea border regions, from 2023 to 2024.

Virus species	Total number (%) ticks positive [95% CI]			
	Helong	Hunchun	Antu	Longjing
Songling virus	7/58 (1.80) [ 0.80–3.56]	4/107 (0.60) [ 0.20–1.45]	7/82 (1.23) [ 0.55–2.39]	14/40 (5.80) [ 3.39–9.41]
Lesnoe mivirus	13/58 (3.40) [ 1.94–5.58]	10/107 (1.49) [ 0.78–2.62]	2/82 (0.35) [ 0.06–1.13]	9/40 (3.37) [ 1.69–6.09]
Xue-Cheng virus	0	2/107 (0.30) [ 0.05–0.96]	0	0
Dabieshan tick virus	0	31/107 (5.77) [ 4.01–8.09]	0	0
Hunchun nairovirus	0	4/107 (0.60) [ 0.19–1.42]	0	0
Ji'an nairovirus	0	2/107 (0.29) [ 0.05–0.96]	0	0
Sara tick phlebovirus	0	1/107 (0.15) [ 0.01–0.72]	2/82 (0.35) [ 0.06–1.14]	0
Yanggou tick virus	0	0	2/82 (0.35) [ 0.06–1.14]	0
Yanbian Rhabd tick virus 1	0	0	1/82 (0.17) [ 0.01–0.84]	0
Yanbian Rhabd tick virus 4	0	0	2/82 (0.35) [ 0.06–1.13]	0
Beiji nairovirus	1/58 (0.24) [ 0.01–1.16]	0	0	0
Mukawa phlebovirus	0	0	0	1/40 (0.34) [ 0.02–1.62]
Manly virus	0	0	2/82 (0.35) [ 0.06–1.13]	0
Tahe rhabdovirus 1	0	0	2/82 (0.35) [ 0.06–1.13]	0

*Note:* The rates at which ticks were infected with viruses were calculated using the bias-corrected MLE method in PooledInfRate software; 95% confidence intervals (CIs) are presented in brackets.

## Data Availability

All sequence reads generated in this project are available in the NCBI Short Read Archive (SRA) under BioProject PRJNA1320731 (https://www.ncbi.nlm.nih.gov/bioproject/PRJNA1320731) with the following accession numbers: SRR35275679–SRR35275682. All generated nucleotide sequences have been deposited in the GenBank database under accession numbers PV641951–PV642101.

## Supporting Information

Supporting Information 1 Grouping and sorting information for tick samples.

Supporting Information 2 Primer sequences used for virus detection in tick samples.

Supporting Information 3 Detailed information on PCR-amplified viral sequences: accession numbers, collection sites, and host species.

Supporting Information 4 Summary of viruses identified from each library.

Supporting Information 5 Agarose gel electrophoresis of RNA viruses in this study.

Supporting Information 6 PCR-based survey of tick-borne viruses infecting humans and animals in the China–Russia–North Korea border region.
