# Supplementary material for: Metatranscriptomic Analysis Uncovers RNA Virus Diversity in Ticks From the China–Russia–North Korea Border Region
Source: Transbound Emerg Dis. 2025 Oct 12;2025:7807512. doi: 10.1155/tbed/7807512 (PMC12535811; doi:10.1155/tbed/7807512)
Supplement: Supporting Information 1 — Grouping and sorting information for tick samples. [file 7807512.f1.docx]

**Supporting Information 1. Grouping and sorting information for tick samples.**

| **Pools name** | **Location** | **Species** | **Life stages** | **Sex** | **Host** | **Ticks number** |
| --- | --- | --- | --- | --- | --- | --- |
| A1 | Antu | *Dermacentor silvarum* | adults | Female | free | 10 |
| A2 | Antu | *Dermacentor silvarum* | adults | Female | free | 10 |
| A3 | Antu | *Dermacentor silvarum* | adults | Female | free | 10 |
| A4 | Antu | *Dermacentor silvarum* | adults | Female | free | 10 |
| A5 | Antu | *Dermacentor silvarum* | adults | Female | free | 10 |
| A6 | Antu | *Dermacentor silvarum* | adults | Female | free | 10 |
| A7 | Antu | *Dermacentor silvarum* | adults | Female | free | 10 |
| A8 | Antu | *Dermacentor silvarum* | adults | Female | free | 10 |
| A9 | Antu | *Dermacentor silvarum* | adults | Female | free | 10 |
| A10 | Antu | *Dermacentor silvarum* | adults | Female | free | 10 |
| A11 | Antu | *Dermacentor silvarum* | adults | Female | free | 10 |
| A12 | Antu | *Dermacentor silvarum* | adults | Female | free | 10 |
| A13 | Antu | *Dermacentor silvarum* | adults | Female | free | 10 |
| A14 | Antu | *Dermacentor silvarum* | adults | Female | free | 10 |
| A15 | Antu | *Dermacentor silvarum* | adults | Female | free | 10 |
| A16 | Antu | *Dermacentor silvarum* | adults | Female | free | 10 |
| A17 | Antu | *Dermacentor silvarum* | adults | Female | free | 10 |
| A18 | Antu | *Dermacentor silvarum* | adults | Female | free | 10 |
| A19 | Antu | *Dermacentor silvarum* | adults | Female | free | 9 |
| A20 | Antu | *Dermacentor silvarum* | adults | Female | free | 9 |
| A21 | Antu | *Dermacentor silvarum* | adults | Female | free | 8 |
| A22 | Antu | *Dermacentor silvarum* | adults | Female | free | 7 |
| A23 | Antu | *Dermacentor silvarum* | adults | Female | free | 6 |
| A24 | Antu | *Dermacentor silvarum* | adults | Female | free | 1 |
| A25 | Antu | *Dermacentor silvarum* | adults | Male | free | 11 |
| A26 | Antu | *Dermacentor silvarum* | adults | Male | free | 10 |
| A27 | Antu | *Dermacentor silvarum* | adults | Male | free | 10 |
| A28 | Antu | *Dermacentor silvarum* | adults | Male | free | 10 |
| A29 | Antu | *Dermacentor silvarum* | adults | Male | free | 10 |
| A30 | Antu | *Dermacentor silvarum* | adults | Male | free | 10 |
| A31 | Antu | *Dermacentor silvarum* | adults | Male | free | 10 |
| A32 | Antu | *Dermacentor silvarum* | adults | Male | free | 10 |
| A33 | Antu | *Dermacentor silvarum* | adults | Male | free | 9 |
| A34 | Antu | *Dermacentor silvarum* | adults | Male | free | 9 |
| A35 | Antu | *Dermacentor silvarum* | adults | Male | free | 6 |
| A36 | Antu | *Dermacentor silvarum* | adults | Male | free | 8 |
| A37 | Antu | *Dermacentor silvarum* | adults | Male | free | 7 |
| A38 | Antu | *Dermacentor silvarum* | adults | Male | free | 6 |
| A39 | Antu | *Dermacentor silvarum* | adults | Male | free | 5 |
| A40 | Antu | *Dermacentor silvarum* | adults | Male | free | 5 |
| A41 | Antu | *Dermacentor silvarum* | adults | Male | free | 5 |
| A42 | Antu | *Dermacentor silvarum* | adults | Male | free | 5 |
| A43 | Antu | *Dermacentor silvarum* | adults | Male | free | 5 |
| A44 | Antu | *Dermacentor silvarum* | adults | Male | free | 3 |
| A45 | Antu | *Haemaphysalis concinna* | adults | Female | free | 11 |
| A46 | Antu | *Haemaphysalis concinna* | adults | Female | free | 10 |
| A47 | Antu | *Haemaphysalis concinna* | adults | Female | free | 10 |
| A48 | Antu | *Haemaphysalis concinna* | adults | Female | free | 10 |
| A49 | Antu | *Haemaphysalis concinna* | adults | Female | free | 8 |
| A50 | Antu | *Haemaphysalis concinna* | adults | Female | free | 4 |
| A51 | Antu | *Haemaphysalis concinna* | adults | Female | free | 3 |
| A52 | Antu | *Haemaphysalis concinna* | adults | Female | free | 1 |
| A53 | Antu | *Haemaphysalis concinna* | adults | Male | free | 10 |
| A54 | Antu | *Haemaphysalis concinna* | adults | Male | free | 10 |
| A55 | Antu | *Haemaphysalis concinna* | adults | Male | free | 10 |
| A56 | Antu | *Haemaphysalis concinna* | adults | Male | free | 9 |
| A57 | Antu | *Haemaphysalis concinna* | adults | Male | free | 8 |
| A58 | Antu | *Haemaphysalis concinna* | adults | Male | free | 7 |
| A59 | Antu | *Haemaphysalis concinna* | adults | Male | free | 1 |
| A60 | Antu | *Haemaphysalis concinna* | adults | Male | free | 1 |
| A61 | Antu | *Haemaphysalis concinna* | adults | Male | free | 1 |
| A62 | Antu | *Haemaphysalis japonica* | adults | Female | free | 10 |
| A63 | Antu | *Haemaphysalis japonica* | adults | Female | free | 10 |
| A64 | Antu | *Haemaphysalis japonica* | adults | Female | free | 9 |
| A65 | Antu | *Haemaphysalis japonica* | adults | Female | free | 7 |
| A66 | Antu | *Haemaphysalis japonica* | adults | Female | free | 5 |
| A67 | Antu | *Haemaphysalis japonica* | adults | Female | free | 5 |
| A68 | Antu | *Haemaphysalis japonica* | adults | Female | free | 1 |
| A69 | Antu | *Haemaphysalis japonica* | adults | Male | free | 4 |
| A70 | Antu | *Haemaphysalis japonica* | adults | Male | free | 4 |
| A71 | Antu | *Haemaphysalis japonica* | adults | Male | free | 3 |
| A72 | Antu | *Haemaphysalis japonica* | adults | Male | free | 2 |
| A73 | Antu | *Haemaphysalis japonica* | adults | Male | free | 2 |
| A74 | Antu | *Ixodes persulcatus* | adults | Female | free | 13 |
| A75 | Antu | *Ixodes persulcatus* | adults | Female | free | 3 |
| A76 | Antu | *Ixodes persulcatus* | adults | Female | free | 2 |
| A77 | Antu | *Ixodes persulcatus* | adults | Female | free | 1 |
| A78 | Antu | *Ixodes persulcatus* | adults | Female | free | 1 |
| A79 | Antu | *Ixodes persulcatus* | adults | Male | free | 3 |
| A80 | Antu | *Ixodes persulcatus* | adults | Male | free | 3 |
| A81 | Antu | *Ixodes persulcatus* | adults | Male | free | 1 |
| A82 | Antu | *Ixodes persulcatus* | adults | Male | free | 1 |
| B1 | Hunchun | *Dermacentor silvarum* | adults | Female | free | 11 |
| B2 | Hunchun | *Dermacentor silvarum* | adults | Female | free | 7 |
| B3 | Hunchun | *Dermacentor silvarum* | adults | Female | free | 2 |
| B4 | Hunchun | *Dermacentor silvarum* | adults | Female | free | 1 |
| B5 | Hunchun | *Dermacentor silvarum* | adults | Female | free | 1 |
| B6 | Hunchun | *Dermacentor silvarum* | adults | Female | free | 1 |
| B7 | Hunchun | *Dermacentor silvarum* | adults | Female | free | 1 |
| B8 | Hunchun | *Dermacentor silvarum* | adults | Female | free | 1 |
| B9 | Hunchun | *Dermacentor silvarum* | adults | Male | free | 4 |
| B10 | Hunchun | *Dermacentor silvarum* | adults | Male | free | 3 |
| B11 | Hunchun | *Dermacentor silvarum* | adults | Male | free | 2 |
| B12 | Hunchun | *Haemaphysalis concinna* | adults | Female | free | 8 |
| B13 | Hunchun | *Haemaphysalis concinna* | adults | Female | free | 7 |
| B14 | Hunchun | *Haemaphysalis concinna* | adults | Female | free | 4 |
| B15 | Hunchun | *Haemaphysalis concinna* | adults | Female | free | 1 |
| B16 | Hunchun | *Haemaphysalis concinna* | adults | Female | free | 1 |
| B17 | Hunchun | *Haemaphysalis concinna* | adults | Female | free | 1 |
| B18 | Hunchun | *Haemaphysalis concinna* | adults | Female | free | 1 |
| B19 | Hunchun | *Haemaphysalis concinna* | adults | Male | free | 1 |
| B20 | Hunchun | *Haemaphysalis concinna* | adults | Male | free | 1 |
| B21 | Hunchun | *Haemaphysalis concinna* | adults | Male | free | 1 |
| B22 | Hunchun | *Haemaphysalis concinna* | adults | Male | free | 1 |
| B23 | Hunchun | *Haemaphysalis longicornis* | adults | Female | free | 11 |
| B24 | Hunchun | *Haemaphysalis longicornis* | adults | Female | free | 10 |
| B25 | Hunchun | *Haemaphysalis longicornis* | adults | Female | free | 10 |
| B26 | Hunchun | *Haemaphysalis longicornis* | adults | Female | free | 10 |
| B27 | Hunchun | *Haemaphysalis longicornis* | adults | Female | free | 10 |
| B28 | Hunchun | *Haemaphysalis longicornis* | adults | Female | free | 10 |
| B29 | Hunchun | *Haemaphysalis longicornis* | adults | Female | free | 10 |
| B30 | Hunchun | *Haemaphysalis longicornis* | adults | Female | free | 10 |
| B31 | Hunchun | *Haemaphysalis longicornis* | adults | Female | free | 10 |
| B32 | Hunchun | *Haemaphysalis longicornis* | adults | Female | free | 10 |
| B33 | Hunchun | *Haemaphysalis longicornis* | adults | Female | free | 10 |
| B34 | Hunchun | *Haemaphysalis longicornis* | adults | Female | free | 10 |
| B35 | Hunchun | *Haemaphysalis longicornis* | adults | Female | free | 10 |
| B36 | Hunchun | *Haemaphysalis longicornis* | adults | Female | free | 10 |
| B37 | Hunchun | *Haemaphysalis longicornis* | adults | Female | free | 10 |
| B38 | Hunchun | *Haemaphysalis longicornis* | adults | Female | free | 10 |
| B39 | Hunchun | *Haemaphysalis longicornis* | adults | Female | free | 10 |
| B40 | Hunchun | *Haemaphysalis longicornis* | adults | Female | free | 10 |
| B41 | Hunchun | *Haemaphysalis longicornis* | adults | Female | free | 10 |
| B42 | Hunchun | *Haemaphysalis longicornis* | adults | Female | free | 10 |
| B43 | Hunchun | *Haemaphysalis longicornis* | adults | Female | free | 7 |
| B44 | Hunchun | *Haemaphysalis longicornis* | adults | Female | free | 6 |
| B45 | Hunchun | *Haemaphysalis longicornis* | adults | Female | free | 6 |
| B46 | Hunchun | *Haemaphysalis longicornis* | adults | Female | free | 2 |
| B47 | Hunchun | *Haemaphysalis longicornis* | adults | Female | free | 2 |
| B48 | Hunchun | *Haemaphysalis longicornis* | adults | Female | free | 2 |
| B49 | Hunchun | *Haemaphysalis longicornis* | adults | Female | free | 1 |
| B50 | Hunchun | *Haemaphysalis longicornis* | adults | Male | free | 11 |
| B51 | Hunchun | *Haemaphysalis longicornis* | adults | Male | free | 11 |
| B52 | Hunchun | *Haemaphysalis longicornis* | adults | Male | free | 11 |
| B53 | Hunchun | *Haemaphysalis longicornis* | adults | Male | free | 10 |
| B54 | Hunchun | *Haemaphysalis longicornis* | adults | Male | free | 10 |
| B55 | Hunchun | *Haemaphysalis longicornis* | adults | Male | free | 10 |
| B56 | Hunchun | *Haemaphysalis longicornis* | adults | Male | free | 10 |
| B57 | Hunchun | *Haemaphysalis longicornis* | adults | Male | free | 10 |
| B58 | Hunchun | *Haemaphysalis longicornis* | adults | Male | free | 10 |
| B59 | Hunchun | *Haemaphysalis longicornis* | adults | Male | free | 10 |
| B60 | Hunchun | *Haemaphysalis longicornis* | adults | Male | free | 10 |
| B61 | Hunchun | *Haemaphysalis longicornis* | adults | Male | free | 10 |
| B62 | Hunchun | *Haemaphysalis longicornis* | adults | Male | free | 10 |
| B63 | Hunchun | *Haemaphysalis longicornis* | adults | Male | free | 10 |
| B64 | Hunchun | *Haemaphysalis longicornis* | adults | Male | free | 10 |
| B65 | Hunchun | *Haemaphysalis longicornis* | adults | Male | free | 10 |
| B66 | Hunchun | *Haemaphysalis longicornis* | adults | Male | free | 10 |
| B67 | Hunchun | *Haemaphysalis longicornis* | adults | Male | free | 10 |
| B68 | Hunchun | *Haemaphysalis longicornis* | adults | Male | free | 10 |
| B69 | Hunchun | *Haemaphysalis longicornis* | adults | Male | free | 10 |
| B70 | Hunchun | *Haemaphysalis longicornis* | adults | Male | free | 10 |
| B71 | Hunchun | *Haemaphysalis longicornis* | adults | Male | free | 10 |
| B72 | Hunchun | *Haemaphysalis longicornis* | adults | Male | free | 10 |
| B73 | Hunchun | *Haemaphysalis longicornis* | adults | Male | free | 8 |
| B74 | Hunchun | *Haemaphysalis longicornis* | adults | Male | free | 7 |
| B75 | Hunchun | *Haemaphysalis longicornis* | adults | Male | free | 6 |
| B76 | Hunchun | *Haemaphysalis longicornis* | adults | Male | free | 4 |
| B77 | Hunchun | *Haemaphysalis longicornis* | adults | Male | free | 2 |
| B78 | Hunchun | *Haemaphysalis longicornis* | adults | Male | free | 2 |
| B79 | Hunchun | *Haemaphysalis longicornis* | adults | Male | free | 2 |
| B80 | Hunchun | *Haemaphysalis longicornis* | adults | Male | free | 1 |
| B81 | Hunchun | *Haemaphysalis longicornis* | adults | Male | free | 1 |
| B82 | Hunchun | *Haemaphysalis japonica* | adults | Female | free | 5 |
| B83 | Hunchun | *Haemaphysalis japonica* | adults | Female | free | 3 |
| B84 | Hunchun | *Haemaphysalis japonica* | adults | Female | free | 2 |
| B85 | Hunchun | *Haemaphysalis japonica* | adults | Female | free | 2 |
| B86 | Hunchun | *Haemaphysalis japonica* | adults | Female | free | 1 |
| B87 | Hunchun | *Haemaphysalis japonica* | adults | Female | free | 1 |
| B88 | Hunchun | *Haemaphysalis japonica* | adults | Male | free | 7 |
| B89 | Hunchun | *Haemaphysalis japonica* | adults | Male | free | 5 |
| B90 | Hunchun | *Haemaphysalis japonica* | adults | Male | free | 4 |
| B91 | Hunchun | *Haemaphysalis japonica* | adults | Male | free | 4 |
| B92 | Hunchun | *Haemaphysalis japonica* | adults | Male | free | 3 |
| B93 | Hunchun | *Ixodes persulcatus* | adults | Female | free | 16 |
| B94 | Hunchun | *Ixodes persulcatus* | adults | Female | free | 10 |
| B95 | Hunchun | *Ixodes persulcatus* | adults | Female | free | 6 |
| B96 | Hunchun | *Ixodes persulcatus* | adults | Female | free | 3 |
| B97 | Hunchun | *Ixodes persulcatus* | adults | Female | free | 2 |
| B98 | Hunchun | *Ixodes persulcatus* | adults | Female | free | 1 |
| B99 | Hunchun | *Ixodes persulcatus* | adults | Female | free | 1 |
| B100 | Hunchun | *Ixodes persulcatus* | adults | Male | free | 11 |
| B101 | Hunchun | *Ixodes persulcatus* | adults | Male | free | 8 |
| B102 | Hunchun | *Ixodes persulcatus* | adults | Male | free | 7 |
| B103 | Hunchun | *Ixodes persulcatus* | adults | Male | free | 6 |
| B104 | Hunchun | *Ixodes persulcatus* | adults | Male | free | 6 |
| B105 | Hunchun | *Ixodes persulcatus* | adults | Male | free | 4 |
| B106 | Hunchun | *Ixodes persulcatus* | adults | Male | free | 2 |
| B107 | Hunchun | *Ixodes persulcatus* | adults | Male | free | 2 |
| C1 | Helong | *Haemaphysalis concinna* | adults | Female | free | 10 |
| C2 | Helong | *Haemaphysalis concinna* | adults | Female | free | 10 |
| C3 | Helong | *Haemaphysalis concinna* | adults | Female | free | 10 |
| C4 | Helong | *Haemaphysalis concinna* | adults | Female | free | 10 |
| C5 | Helong | *Haemaphysalis concinna* | adults | Female | free | 10 |
| C6 | Helong | *Haemaphysalis concinna* | adults | Female | free | 10 |
| C7 | Helong | *Haemaphysalis concinna* | adults | Female | free | 10 |
| C8 | Helong | *Haemaphysalis concinna* | adults | Female | free | 10 |
| C9 | Helong | *Haemaphysalis concinna* | adults | Female | free | 10 |
| C10 | Helong | *Haemaphysalis concinna* | adults | Female | free | 10 |
| C11 | Helong | *Haemaphysalis concinna* | adults | Female | free | 10 |
| C12 | Helong | *Haemaphysalis concinna* | adults | Female | free | 10 |
| C13 | Helong | *Haemaphysalis concinna* | adults | Female | free | 10 |
| C14 | Helong | *Haemaphysalis concinna* | adults | Female | free | 9 |
| C15 | Helong | *Haemaphysalis concinna* | adults | Female | free | 9 |
| C16 | Helong | *Haemaphysalis concinna* | adults | Female | free | 9 |
| C17 | Helong | *Haemaphysalis concinna* | adults | Female | free | 8 |
| C18 | Helong | *Haemaphysalis concinna* | adults | Female | free | 4 |
| C19 | Helong | *Haemaphysalis concinna* | adults | Female | free | 3 |
| C20 | Helong | *Haemaphysalis concinna* | adults | Female | free | 2 |
| C21 | Helong | *Haemaphysalis concinna* | adults | Male | free | 10 |
| C22 | Helong | *Haemaphysalis concinna* | adults | Male | free | 10 |
| C23 | Helong | *Haemaphysalis concinna* | adults | Male | free | 10 |
| C24 | Helong | *Haemaphysalis concinna* | adults | Male | free | 10 |
| C25 | Helong | *Haemaphysalis concinna* | adults | Male | free | 10 |
| C26 | Helong | *Haemaphysalis concinna* | adults | Male | free | 10 |
| C27 | Helong | *Haemaphysalis concinna* | adults | Male | free | 10 |
| C28 | Helong | *Haemaphysalis concinna* | adults | Male | free | 10 |
| C29 | Helong | *Haemaphysalis concinna* | adults | Male | free | 10 |
| C30 | Helong | *Haemaphysalis concinna* | adults | Male | free | 10 |
| C31 | Helong | *Haemaphysalis concinna* | adults | Male | free | 10 |
| C32 | Helong | *Haemaphysalis concinna* | adults | Male | free | 10 |
| C33 | Helong | *Haemaphysalis concinna* | adults | Male | free | 10 |
| C34 | Helong | *Haemaphysalis concinna* | adults | Male | free | 10 |
| C35 | Helong | *Haemaphysalis concinna* | adults | Male | free | 10 |
| C36 | Helong | *Haemaphysalis concinna* | adults | Male | free | 10 |
| C37 | Helong | *Haemaphysalis concinna* | adults | Male | free | 9 |
| C38 | Helong | *Haemaphysalis concinna* | adults | Male | free | 8 |
| C39 | Helong | *Haemaphysalis concinna* | adults | Male | free | 5 |
| C40 | Helong | *Haemaphysalis concinna* | adults | Male | free | 1 |
| C41 | Helong | *Haemaphysalis concinna* | adults | Male | free | 1 |
| C42 | Helong | *Haemaphysalis japonica* | adults | Female | free | 4 |
| C43 | Helong | *Haemaphysalis japonica* | adults | Female | free | 2 |
| C44 | Helong | *Haemaphysalis japonica* | adults | Female | free | 1 |
| C45 | Helong | *Haemaphysalis japonica* | adults | Male | free | 2 |
| C46 | Helong | *Haemaphysalis japonica* | adults | Male | free | 2 |
| C47 | Helong | *Haemaphysalis japonica* | adults | Male | free | 1 |
| C48 | Helong | *Ixodes persulcatus* | adults | Female | free | 9 |
| C49 | Helong | *Ixodes persulcatus* | adults | Female | free | 7 |
| C50 | Helong | *Ixodes persulcatus* | adults | Female | free | 7 |
| C51 | Helong | *Ixodes persulcatus* | adults | Female | free | 1 |
| C52 | Helong | *Ixodes persulcatus* | adults | Female | free | 1 |
| C53 | Helong | *Ixodes persulcatus* | adults | Female | free | 1 |
| C54 | Helong | *Ixodes persulcatus* | adults | Female | free | 1 |
| C55 | Helong | *Ixodes persulcatus* | adults | Male | free | 8 |
| C56 | Helong | *Ixodes persulcatus* | adults | Male | free | 5 |
| C57 | Helong | *Ixodes persulcatus* | adults | Male | free | 4 |
| C58 | Helong | *Ixodes persulcatus* | adults | Male | free | 2 |
| D1 | Longjing | *Dermacentor silvarum* | adults | Female | free | 10 |
| D2 | Longjing | *Dermacentor silvarum* | adults | Female | free | 2 |
| D3 | Longjing | *Dermacentor silvarum* | adults | Female | free | 1 |
| D4 | Longjing | *Dermacentor silvarum* | adults | Female | free | 1 |
| D5 | Longjing | *Dermacentor silvarum* | adults | Male | free | 2 |
| D6 | Longjing | *Haemaphysalis concinna* | adults | Female | free | 10 |
| D7 | Longjing | *Haemaphysalis concinna* | adults | Female | free | 10 |
| D8 | Longjing | *Haemaphysalis concinna* | adults | Female | free | 10 |
| D9 | Longjing | *Haemaphysalis concinna* | adults | Female | free | 10 |
| D10 | Longjing | *Haemaphysalis concinna* | adults | Female | free | 10 |
| D11 | Longjing | *Haemaphysalis concinna* | adults | Female | free | 10 |
| D12 | Longjing | *Haemaphysalis concinna* | adults | Female | free | 10 |
| D13 | Longjing | *Haemaphysalis concinna* | adults | Female | free | 10 |
| D14 | Longjing | *Haemaphysalis concinna* | adults | Female | free | 10 |
| D15 | Longjing | *Haemaphysalis concinna* | adults | Female | free | 10 |
| D16 | Longjing | *Haemaphysalis concinna* | adults | Female | free | 10 |
| D17 | Longjing | *Haemaphysalis concinna* | adults | Female | free | 10 |
| D18 | Longjing | *Haemaphysalis concinna* | adults | Female | free | 10 |
| D19 | Longjing | *Haemaphysalis concinna* | adults | Female | free | 7 |
| D20 | Longjing | *Haemaphysalis concinna* | adults | Female | free | 4 |
| D21 | Longjing | *Haemaphysalis concinna* | adults | Female | free | 1 |
| D22 | Longjing | *Haemaphysalis concinna* | adults | Male | free | 10 |
| D23 | Longjing | *Haemaphysalis concinna* | adults | Male | free | 10 |
| D24 | Longjing | *Haemaphysalis concinna* | adults | Male | free | 10 |
| D25 | Longjing | *Haemaphysalis concinna* | adults | Male | free | 10 |
| D26 | Longjing | *Haemaphysalis concinna* | adults | Male | free | 10 |
| D27 | Longjing | *Haemaphysalis concinna* | adults | Male | free | 10 |
| D28 | Longjing | *Haemaphysalis concinna* | adults | Male | free | 10 |
| D29 | Longjing | *Haemaphysalis concinna* | adults | Male | free | 10 |
| D30 | Longjing | *Haemaphysalis concinna* | adults | Male | free | 9 |
| D31 | Longjing | *Haemaphysalis concinna* | adults | Male | free | 2 |
| D32 | Longjing | *Haemaphysalis concinna* | adults | Male | free | 1 |
| D33 | Longjing | *Haemaphysalis japonica* | adults | Female | free | 10 |
| D34 | Longjing | *Haemaphysalis japonica* | adults | Female | free | 10 |
| D35 | Longjing | *Haemaphysalis japonica* | adults | Female | free | 8 |
| D36 | Longjing | *Haemaphysalis japonica* | adults | Female | free | 5 |
| D37 | Longjing | *Haemaphysalis japonica* | adults | Male | free | 3 |
| D38 | Longjing | *Haemaphysalis japonica* | adults | Male | free | 3 |
| D39 | Longjing | *Haemaphysalis japonica* | adults | Male | free | 1 |
| D40 | Longjing | *Ixodes persulcatus* | adults | Female | free | 2 |
