# Supplementary material for: Metatranscriptomic Analysis Uncovers RNA Virus Diversity in Ticks From the China–Russia–North Korea Border Region
Source: Transbound Emerg Dis. 2025 Oct 12;2025:7807512. doi: 10.1155/tbed/7807512 (PMC12535811; doi:10.1155/tbed/7807512)
Supplement: Supporting Information 2 — Primer sequences used for virus detection in tick samples. [file 7807512.f2.docx]

**Supporting Information 2. Primer sequences used for virus detection in tick samples.**

| **For tick identification** | | **Primer sequence** | **Annealing temperature(℃)** | **Amplication size(bp)** |
| --- | --- | --- | --- | --- |
| COI-F  COI-R | | GGTCAACAAATCATAAAGATATTGG  TAAACTTCAGGGTGACCAAAAATCA | **57** | 710 |
| **Family** | **Virus name** | **Primer sequence** | **Annealing temperature(℃)** | **Amplication size(bp)** |
| *Flaviviridae* | Yanggou tick virus | ACTACTGGTTGCCGTCCTCG | 58 | 305 |
|  |  | GTCGCTGCAGTCAAATATCT |  |  |
| *Nairoviridae* | Songling virus | CCCTCTGCACAGGAACAAA | 56 | 500 |
|  |  | GACCCTGTAGATCTTGACAAC |  |  |
|  | Beiji nairovirus | CCGTTTACCCAGCCTACC | 58 | 498 |
|  |  | TGTGGTGTTCCCTGAAGTTAGT |  |  |
|  | Hunchun nairovirus | ACATAATGAGCAATCTCCTG | 54 | 457 |
|  |  | CTCTTCTAGGACAGCAATGG |  |  |
|  | Ji'an nairovirus | TTGTGGCTCAGTCCAAAGAT | 57 | 1375 |
|  |  | GAAATGCTCTACCCCTCCTC |  |  |
|  | Xue-Cheng virus | GGAGACCGTACCAAATGGGG | 57 | 766 |
|  |  | CCTGAACCATGCTCTGTGGT |  |  |
| *Phenuiviridae* | Dabieshan tick virus | CTGGACCAAGGATGTGGCAT | 60 | 520 |
|  |  | CAGCTTCTTGAGGCTCTGCT |  |  |
|  | Sara tick phlebovirus | AGAGCCTGCCGAGAACAG | 56 | 443 |
|  |  | GAATCCCGTAGTGCTTGACC |  |  |
|  | Mukawa phlebovirus | CCATCAATCTGTACACCAGG | 56 | 895 |
|  |  | ACACAAAGTCCGCCCATTAC |  |  |
| *Chuviridae* | Lesnoe mivirus | GGAAATGCGGAGAACTTGA | 56 | 1281 |
|  |  | CTGGTGCCACTGATGCTG |  |  |
| *Rhabdoviridae* | Tahe rhabdovirus 1 | GAGTGATGGGTCAGTTCTTCG | 57 | 239 |
|  |  | AACCTTGGCTTCCCTGAGTA |  |  |
|  | Yanbian Rhabd tick virus 4 | AGCGGAAGAGAGTCCTGGAT | 59 | 579 |
|  |  | GGTCCCTTGGGGTAATTGGG |  |  |
|  | Yanbian Rhabd tick virus 1 | ACTTGAAAGAGCGTGCCG | 56 | 822 |
|  |  | ACCCAGCCTCCAATCGT |  |  |
|  | Manly virus | ATCTGTGGAGCAAGAGTAGTGG | 57 | 764 |
|  |  | CTTCGCATAATCCTCGTCAA |  |  |
| *Tombusviridae* | Cheeloo tick virus 3 | TCTGCGGAGACTGCGTTAG | 57 | 1436 |
|  |  | CACTTCAGTGGCGAGGATG |  |  |
|  | Ningxia luteovirus | GGATGGCTCGGAGGACA | 57 | 1439 |
|  |  | AAAGTAGCACGGGAACGAA |  |  |
| *Partitiviridae* | Jilin partiti-like virus 1 | CCGCCCCACAAAGAACA | 57 | 345 |
|  |  | CCCGCTGAAGAGGTAGGAA |  |  |
| *Solemoviridae* | Xinjiang tick associated virus 1 | ACCGATTCCGTCAATGTCC | 57 | 754 |
|  |  | CAGCATCGTAGCCTATGTTTTC |  |  |
|  | Ixodes scapularis associated virus 1 | GGGTGCGTTTGGTGGAC | 57 | 324 |
|  |  | CAACAGGACGAAACCGATG |  |  |
|  | Hubei sobemo-like virus 15 | GGTGTTAGCCTACTGGTCGTT | 57 | 534 |
|  |  | CCTTTCCCGCACTTCATG |  |  |
| *Hepeviridae* | Hepelivirales sp. | CACCCTGGGATTATTCGG | 56 | 879 |
|  |  | AGCTCTTTGAGTTGCTTTGC |  |  |
