# Supplementary material for: Metatranscriptomic Analysis Uncovers RNA Virus Diversity in Ticks From the China–Russia–North Korea Border Region
Source: Transbound Emerg Dis. 2025 Oct 12;2025:7807512. doi: 10.1155/tbed/7807512 (PMC12535811; doi:10.1155/tbed/7807512)
Supplement: Supporting Information 3 — Detailed information on PCR-amplified viral sequences: accession numbers, collection sites, and host species. [file 7807512.f3.docx]

**Supporting Information 3. Detailed information on PCR-amplified viral sequences: Accession numbers, collection sites and host species.**

| **Viral species** | **Pools name** | **Collection sites** | **Host species** | **GenBank accession NO.** |
| --- | --- | --- | --- | --- |
| Lesnoe mivirus | C1 | Helong | *Haemaphysalis concinna* | PV641990 |
| Lesnoe mivirus | C8 | Helong | *Haemaphysalis concinna* | PV641991 |
| Lesnoe mivirus | C17 | Helong | *Haemaphysalis concinna* | PV641992 |
| Lesnoe mivirus | C19 | Helong | *Haemaphysalis concinna* | PV641993 |
| Lesnoe mivirus | C22 | Helong | *Haemaphysalis concinna* | PV641994 |
| Lesnoe mivirus | C26 | Helong | *Haemaphysalis concinna* | PV641995 |
| Lesnoe mivirus | C27 | Helong | *Haemaphysalis concinna* | PV641996 |
| Lesnoe mivirus | C28 | Helong | *Haemaphysalis concinna* | PV641997 |
| Lesnoe mivirus | C39 | Helong | *Haemaphysalis concinna* | PV641998 |
| Lesnoe mivirus | C42 | Helong | *Haemaphysalis japonica* | PV641999 |
| Lesnoe mivirus | C45 | Helong | *Haemaphysalis japonica* | PV642000 |
| Lesnoe mivirus | C46 | Helong | *Haemaphysalis japonica* | PV642001 |
| Lesnoe mivirus | C47 | Helong | *Haemaphysalis japonica* | PV642002 |
| Lesnoe mivirus | B12 | Hunchun | *Haemaphysalis concinna* | PV642005 |
| Lesnoe mivirus | B13 | Hunchun | *Haemaphysalis concinna* | PV642006 |
| Lesnoe mivirus | B15 | Hunchun | *Haemaphysalis concinna* | PV642007 |
| Lesnoe mivirus | B19 | Hunchun | *Haemaphysalis concinna* | PV642008 |
| Lesnoe mivirus | B20 | Hunchun | *Haemaphysalis concinna* | PV642009 |
| Lesnoe mivirus | B21 | Hunchun | *Haemaphysalis concinna* | PV642010 |
| Lesnoe mivirus | B83 | Hunchun | *Haemaphysalis japonica* | PV642011 |
| Lesnoe mivirus | B87 | Hunchun | *Haemaphysalis japonica* | PV642012 |
| Lesnoe mivirus | B91 | Hunchun | *Haemaphysalis japonica* | PV642013 |
| Lesnoe mivirus | B92 | Hunchun | *Haemaphysalis japonica* | PV642014 |
| Lesnoe mivirus | D2 | Longjing | *Dermacentor silvarum* | PV641981 |
| Lesnoe mivirus | D8 | Longjing | *Haemaphysalis concinna* | PV641982 |
| Lesnoe mivirus | D9 | Longjing | *Haemaphysalis concinna* | PV641983 |
| Lesnoe mivirus | D11 | Longjing | *Haemaphysalis concinna* | PV641984 |
| Lesnoe mivirus | D18 | Longjing | *Haemaphysalis concinna* | PV641985 |
| Lesnoe mivirus | D19 | Longjing | *Haemaphysalis concinna* | PV641986 |
| Lesnoe mivirus | D20 | Longjing | *Haemaphysalis concinna* | PV641987 |
| Lesnoe mivirus | D33 | Longjing | *Haemaphysalis japonica* | PV641988 |
| Lesnoe mivirus | D39 | Longjing | *Haemaphysalis japonica* | PV641989 |
| Lesnoe mivirus | A37 | Antu | *Dermacentor silvarum* | PV642003 |
| Lesnoe mivirus | A70 | Antu | *Haemaphysalis japonica* | PV642004 |
| Songling virus | D6 | Longjing | *Haemaphysalis concinna* | PV642059 |
| Songling virus | D7 | Longjing | *Haemaphysalis concinna* | PV642060 |
| Songling virus | D12 | Longjing | *Haemaphysalis concinna* | PV642061 |
| Songling virus | D15 | Longjing | *Haemaphysalis concinna* | PV642062 |
| Songling virus | D16 | Longjing | *Haemaphysalis concinna* | PV642063 |
| Songling virus | D17 | Longjing | *Haemaphysalis concinna* | PV642064 |
| Songling virus | D21 | Longjing | *Haemaphysalis concinna* | PV642065 |
| Songling virus | D22 | Longjing | *Haemaphysalis concinna* | PV642066 |
| Songling virus | D24 | Longjing | *Haemaphysalis concinna* | PV642067 |
| Songling virus | D27 | Longjing | *Haemaphysalis concinna* | PV642068 |
| Songling virus | D29 | Longjing | *Haemaphysalis concinna* | PV642069 |
| Songling virus | D30 | Longjing | *Haemaphysalis concinna* | PV642070 |
| Songling virus | D31 | Longjing | *Haemaphysalis concinna* | PV642071 |
| Songling virus | D32 | Longjing | *Haemaphysalis concinna* | PV642072 |
| Songling virus | C5 | Helong | *Haemaphysalis concinna* | PV642052 |
| Songling virus | C16 | Helong | *Haemaphysalis concinna* | PV642053 |
| Songling virus | C23 | Helong | *Haemaphysalis concinna* | PV642054 |
| Songling virus | C24 | Helong | *Haemaphysalis concinna* | PV642055 |
| Songling virus | C29 | Helong | *Haemaphysalis concinna* | PV642056 |
| Songling virus | C30 | Helong | *Haemaphysalis concinna* | PV642057 |
| Songling virus | C33 | Helong | *Haemaphysalis concinna* | PV642058 |
| Songling virus | A49 | Antu | *Haemaphysalis concinna* | PV642073 |
| Songling virus | A52 | Antu | *Haemaphysalis concinna* | PV642074 |
| Songling virus | A55 | Antu | *Haemaphysalis concinna* | PV642075 |
| Songling virus | A58 | Antu | *Haemaphysalis concinna* | PV642076 |
| Songling virus | A60 | Antu | *Haemaphysalis concinna* | PV642077 |
| Songling virus | A76 | Antu | *Ixodes persulcatus* | PV642078 |
| Songling virus | A81 | Antu | *Ixodes persulcatus* | PV642079 |
| Songling virus | B42 | Hunchun | *Haemaphysalis longicornis* | PV642081 |
| Songling virus | B70 | Hunchun | *Haemaphysalis longicornis* | PV642082 |
| Songling virus | B71 | Hunchun | *Haemaphysalis longicornis* | PV642083 |
| Songling virus | B100 | Hunchun | *Ixodes persulcatus* | PV642080 |
| Dabieshan tick virus | B23 | Hunchun | *Haemaphysalis longicornis* | PV642021 |
| Dabieshan tick virus | B26 | Hunchun | *Haemaphysalis longicornis* | PV642022 |
| Dabieshan tick virus | B27 | Hunchun | *Haemaphysalis longicornis* | PV642023 |
| Dabieshan tick virus | B29 | Hunchun | *Haemaphysalis longicornis* | PV642024 |
| Dabieshan tick virus | B30 | Hunchun | *Haemaphysalis longicornis* | PV642025 |
| Dabieshan tick virus | B31 | Hunchun | *Haemaphysalis longicornis* | PV642026 |
| Dabieshan tick virus | B35 | Hunchun | *Haemaphysalis longicornis* | PV642027 |
| Dabieshan tick virus | B36 | Hunchun | *Haemaphysalis longicornis* | PV642028 |
| Dabieshan tick virus | B37 | Hunchun | *Haemaphysalis longicornis* | PV642029 |
| Dabieshan tick virus | B38 | Hunchun | *Haemaphysalis longicornis* | PV642030 |
| Dabieshan tick virus | B39 | Hunchun | *Haemaphysalis longicornis* | PV642031 |
| Dabieshan tick virus | B42 | Hunchun | *Haemaphysalis longicornis* | PV642032 |
| Dabieshan tick virus | B43 | Hunchun | *Haemaphysalis longicornis* | PV642033 |
| Dabieshan tick virus | B44 | Hunchun | *Haemaphysalis longicornis* | PV642034 |
| Dabieshan tick virus | B45 | Hunchun | *Haemaphysalis longicornis* | PV642035 |
| Dabieshan tick virus | B46 | Hunchun | *Haemaphysalis longicornis* | PV642036 |
| Dabieshan tick virus | B51 | Hunchun | *Haemaphysalis longicornis* | PV642037 |
| Dabieshan tick virus | B55 | Hunchun | *Haemaphysalis longicornis* | PV642038 |
| Dabieshan tick virus | B56 | Hunchun | *Haemaphysalis longicornis* | PV642039 |
| Dabieshan tick virus | B57 | Hunchun | *Haemaphysalis longicornis* | PV642040 |
| Dabieshan tick virus | B59 | Hunchun | *Haemaphysalis longicornis* | PV642041 |
| Dabieshan tick virus | B60 | Hunchun | *Haemaphysalis longicornis* | PV642042 |
| Dabieshan tick virus | B61 | Hunchun | *Haemaphysalis longicornis* | PV642043 |
| Dabieshan tick virus | B62 | Hunchun | *Haemaphysalis longicornis* | PV642044 |
| Dabieshan tick virus | B63 | Hunchun | *Haemaphysalis longicornis* | PV642045 |
| Dabieshan tick virus | B67 | Hunchun | *Haemaphysalis longicornis* | PV642046 |
| Dabieshan tick virus | B68 | Hunchun | *Haemaphysalis longicornis* | PV642047 |
| Dabieshan tick virus | B70 | Hunchun | *Haemaphysalis longicornis* | PV642048 |
| Dabieshan tick virus | B71 | Hunchun | *Haemaphysalis longicornis* | PV642049 |
| Dabieshan tick virus | B72 | Hunchun | *Haemaphysalis longicornis* | PV642050 |
| Dabieshan tick virus | B75 | Hunchun | *Haemaphysalis longicornis* | PV642051 |
| Xinjiang tick associated virus 1 | A20 | Antu | *Dermacentor silvarum* | PV641964 |
| Xinjiang tick associated virus 1 | A5 | Antu | *Dermacentor silvarum* | PV641966 |
| Xinjiang tick associated virus 1 | A16 | Antu | *Dermacentor silvarum* | PV641968 |
| Xinjiang tick associated virus 1 | A22 | Antu | *Dermacentor silvarum* | PV641969 |
| Xinjiang tick associated virus 1 | A25 | Antu | *Dermacentor silvarum* | PV641965 |
| Xinjiang tick associated virus 1 | A36 | Antu | *Dermacentor silvarum* | PV641967 |
| Xinjiang tick associated virus 1 | D1 | Longjing | *Dermacentor silvarum* | PV641971 |
| Xinjiang tick associated virus 1 | D5 | Longjing | *Dermacentor silvarum* | PV641972 |
| Xinjiang tick associated virus 1 | B1 | Hunchun | *Dermacentor silvarum* | PV641970 |
| Cheeloo tick virus 3 | B37 | Hunchun | *Haemaphysalis longicornis* | PV642098 |
| Cheeloo tick virus 3 | B39 | Hunchun | *Haemaphysalis longicornis* | PV642097 |
| Cheeloo tick virus 3 | B43 | Hunchun | *Haemaphysalis longicornis* | PV642096 |
| Cheeloo tick virus 3 | B45 | Hunchun | *Haemaphysalis longicornis* | PV642095 |
| Cheeloo tick virus 3 | B62 | Hunchun | *Haemaphysalis longicornis* | PV642099 |
| Cheeloo tick virus 3 | B65 | Hunchun | *Haemaphysalis longicornis* | PV642100 |
| Cheeloo tick virus 3 | B74 | Hunchun | *Haemaphysalis longicornis* | PV642101 |
| Hubei sobemo-like virus 15 | D2 | Longjing | *Dermacentor silvarum* | PV641976 |
| Hubei sobemo-like virus 15 | D6 | Longjing | *Haemaphysalis concinna* | PV641977 |
| Hubei sobemo-like virus 15 | D34 | Longjing | *Haemaphysalis japonica* | PV641978 |
| Hubei sobemo-like virus 15 | B83 | Hunchun | *Haemaphysalis japonica* | PV641973 |
| Hubei sobemo-like virus 15 | B39 | Hunchun | *Haemaphysalis longicornis* | PV641974 |
| Hubei sobemo-like virus 15 | B42 | Hunchun | *Haemaphysalis longicornis* | PV641975 |
| Hepelivirales sp. | B37 | Hunchun | *Haemaphysalis longicornis* | PV642093 |
| Hepelivirales sp. | B38 | Hunchun | *Haemaphysalis longicornis* | PV642092 |
| Hepelivirales sp. | B39 | Hunchun | *Haemaphysalis longicornis* | PV642089 |
| Hepelivirales sp. | B45 | Hunchun | *Haemaphysalis longicornis* | PV642091 |
| Hepelivirales sp. | B51 | Hunchun | *Haemaphysalis longicornis* | PV642090 |
| Hepelivirales sp. | B71 | Hunchun | *Haemaphysalis longicornis* | PV642088 |
| Hunchun nairovirus | B94 | Hunchun | *Ixodes persulcatus* | PV642015 |
| Hunchun nairovirus | B96 | Hunchun | *Ixodes persulcatus* | PV642016 |
| Hunchun nairovirus | B103 | Hunchun | *Ixodes persulcatus* | PV642017 |
| Hunchun nairovirus | B107 | Hunchun | *Ixodes persulcatus* | PV642018 |
| Yanbian Rhabd tick virus 4 | A64 | Antu | *Haemaphysalis japonica* | PV641961 |
| Yanbian Rhabd tick virus 4 | A66 | Antu | *Haemaphysalis japonica* | PV641962 |
| Yanbian Rhabd tick virus 1 | A74 | Antu | *Ixodes persulcatus* | PV641956 |
| Sara tick phlebovirus | A74 | Antu | *Ixodes persulcatus* | PV641951 |
| Sara tick phlebovirus | A75 | Antu | *Ixodes persulcatus* | PV641952 |
| Sara tick phlebovirus | B93 | Hunchun | *Ixodes persulcatus* | PV641953 |
| Xue-Cheng virus | B14 | Hunchun | *Haemaphysalis concinna* | PV642019 |
| Xue-Cheng virus | B92 | Hunchun | *Haemaphysalis japonica* | PV642020 |
| Ji'an nairovirus | B18 | Hunchun | *Haemaphysalis concinna* | PV641979 |
| Ji'an nairovirus | B96 | Hunchun | *Ixodes persulcatus* | PV641980 |
| Yanggou tick virus | A9 | Antu | *Dermacentor silvarum* | PV642084 |
| Yanggou tick virus | A12 | Antu | *Dermacentor silvarum* | PV642085 |
| Manly virus | A9 | Antu | *Dermacentor silvarum* | PV641954 |
| Manly virus | A51 | Antu | *Haemaphysalis concinna* | PV641955 |
| Jilin partiti-like virus 1 | A74 | Antu | *Ixodes persulcatus* | PV641959 |
| Jilin partiti-like virus 1 | B105 | Hunchun | *Ixodes persulcatus* | PV641960 |
| Tahe rhabdovirus 1 | A16 | Antu | *Dermacentor silvarum* | PV641957 |
| Tahe rhabdovirus 1 | A72 | Antu | *Haemaphysalis japonica* | PV641958 |
| Ixodes scapularis associated virus 1 | B105 | Hunchun | *Ixodes persulcatus* | PV641963 |
| Beiji nairovirus | C49 | Helong | *Ixodes persulcatus* | PV642094 |
| Mukawa phlebovirus | D3 | Longjing | *Dermacentor silvarum* | PV642086 |
| Ningxia luteovirus | D34 | Longjing | *Haemaphysalis japonica* | PV642087 |
