# Supplementary material for: Metatranscriptomic Analysis Uncovers RNA Virus Diversity in Ticks From the China–Russia–North Korea Border Region
Source: Transbound Emerg Dis. 2025 Oct 12;2025:7807512. doi: 10.1155/tbed/7807512 (PMC12535811; doi:10.1155/tbed/7807512)
Supplement: Supporting Information 4 — Summary of viruses identified from each library. [file 7807512.f4.docx]

**Supporting Information 4. Summary of viruses identified from each library.**

| **Family** | **Genus** | **Virus (abbreviation)** | **Length (nt)** | **Library** | **Closest relative (accession no)** | **Query Cover** | **Per.Ident** |
| --- | --- | --- | --- | --- | --- | --- | --- |
| *Flaviviridae* | *Jingmenvirus* | YGTV | 2,439 | Antu | Yanggou tick virus isolate YGTV_YBQG1718A (OR148892.1) | 100.00% | 99.67% |
| *Flaviviridae* | *Jingmenvirus* | YGTV | 2,748 | Antu | Yanggou tick virus strain Erzin14-T20074 (MW525322.2) | 100.00% | 96.72% |
| *Nairoviridae* | *Orthonairovirus* | SGLV | 1,464 | Helong、Antu、Longjing | Songling virus strain YC585 segment S (MT328780.1) | 100.00% | 99.45% |
| *Nairoviridae* | *Orthonairovirus* | SGLV | 1,885 | Helong、Hunchun、Antu、Longjing | Songling virus strain YC585 segment S (MT328780.1) | 100.00% | 98.20% |
| *Nairoviridae* | *Orthonairovirus* | SGLV | 1,554 | Helong、Antu、Longjing | Songling virus isolate NE-MEDG5 segment M (PQ475688.1) | 100.00% | 97.42% |
| *Nairoviridae* | *Orthonairovirus* | SGLV | 2,430 | Helong、Antu、Longjing | Songling virus isolate NE-GPLC5 segment M (PQ475695.1) | 99.00% | 97.91% |
| *Nairoviridae* | *Orthonairovirus* | SGLV | 2,505 | Helong、Antu、Longjing | Songling virus isolate NE-MEDG5 segment M (PQ475688.1) | 100.00% | 98.24% |
| *Nairoviridae* | *Orthonairovirus* | SGLV | 2,748 | Helong、Antu、Longjing | Songling virus isolate NE-2AT7 segment M (PQ475691.1) | 100.00% | 98.62% |
| *Nairoviridae* | *Orthonairovirus* | SGLV | 4,335 | Helong、Hunchun、Antu、Longjing | Songling virus isolate NE-2AT7 segment M (PQ475691.1) | 99.00% | 97.84% |
| *Nairoviridae* | *Orthonairovirus* | SGLV | 777 | Helong、Longjing | Songling virus isolate NE-MEDG5 segment L (PQ475679.1) | 92.00% | 99.44% |
| *Nairoviridae* | *Orthonairovirus* | SGLV | 1,122 | Antu、Longjing | Songling virus strain YC585 segment L (MT328779.1) | 99.00% | 98.28% |
| *Nairoviridae* | *Orthonairovirus* | SGLV | 1,539 | Helong、Antu、Longjing | Songling virus strain NE-TH2 segment L (ON408079.1) | 99.00% | 97.70% |
| *Nairoviridae* | *Orthonairovirus* | SGLV | 1,713 | Helong、Antu、Longjing | Songling virus isolate TIGMIC_2 (ON811866.1) | 100.00% | 98.13% |
| *Nairoviridae* | *Orthonairovirus* | SGLV | 1,914 | Helong、Antu、Longjing | Songling virus isolate TIGMIC_3 (ON811868.1) | 100.00% | 96.92% |
| *Nairoviridae* | *Orthonairovirus* | SGLV | 1,986 | Helong、Antu、Longjing | Songling virus strain NE-TH2 segment L (ON408079.1) | 100.00% | 97.78% |
| *Nairoviridae* | *Orthonairovirus* | SGLV | 2,568 | Helong、Antu、Longjing | Songling virus isolate TIGMIC_3 (ON811868.1) | 100.00% | 98.17% |
| *Nairoviridae* | *Orthonairovirus* | SGLV | 3,285 | Helong、Antu、Longjing | Songling virus strain YC585 segment L (MT328779.1) | 100.00% | 98.39% |
| *Nairoviridae* | *Orthonairovirus* | SGLV | 4,689 | Helong、Antu、Longjing | Songling virus isolate TIGMIC_3 (ON811868.1) | 100.00% | 97.50% |
| *Nairoviridae* | *Orthonairovirus* | SGLV | 12,001 | Helong、Hunchun、Antu、Longjing | Songling virus isolate TIGMIC_3 (ON811868.1) | 100.00% | 97.45% |
| *Nairoviridae* | *Orthonairovirus* | BJNV | 525 | Helong | Beiji nairovirus isolate BJNV_YBQG1717 (OR148734.1) | 93.00% | 99.18% |
| *Nairoviridae* | *Orthonairovirus* | HCNV | 693 | Hunchun | Hunchun nairovirus isolate NE-FCH1 segment S (PQ475621.1) | 100.00% | 99.28% |
| *Nairoviridae* | *Orthonairovirus* | HCNV | 1,635 | Hunchun | Hunchun nairovirus isolate NE-FCH1 segment S (PQ475621.1) | 100.00% | 99.82% |
| *Nairoviridae* | *Orthonairovirus* | HCNV | 406 | Hunchun | Hunchun nairovirus isolate NE-FCH1 segment L (PQ475620.1 ) | 100.00% | 99.01% |
| *Nairoviridae* | *Orthonairovirus* | HCNV | 424 | Hunchun | Hunchun nairovirus isolate NE-FCH1 segment L (PQ475620.1 ) | 98.00% | 99.28% |
| *Nairoviridae* | *Orthonairovirus* | HCNV | 444 | Hunchun | Hunchun nairovirus isolate NE-FCH1 segment L (PQ475620.1) | 95.00% | 99.53% |
| *Nairoviridae* | *Orthonairovirus* | HCNV | 534 | Hunchun | Hunchun nairovirus isolate NE-FCH1 segment L (PQ475620.1) | 98.00% | 99.81% |
| *Nairoviridae* | *Orthonairovirus* | HCNV | 696 | Hunchun | Hunchun nairovirus isolate NE-FCH1 segment L (PQ475620.1) | 97.00% | 99.26% |
| *Nairoviridae* | *Orthonairovirus* | HCNV | 708 | Hunchun | Hunchun nairovirus isolate NE-FCH1 segment L (PQ475620.1) | 98.00% | 99.28% |
| *Nairoviridae* | *Orthonairovirus* | HCNV | 1,284 | Hunchun | Hunchun nairovirus isolate NE-FCH1 segment L (PQ475620.1) | 100.00% | 98.99% |
| *Nairoviridae* | *Orthonairovirus* | JANV | 339 | Hunchun | Ji'an nairovirus isolate NE-LSZ3 segment L (PQ475652.1) | 93.00% | 99.32% |
| *Nairoviridae* | *Orthonairovirus* | JANV | 600 | Hunchun | Ji'an nairovirus isolate NE-DGZ3 segment L (PQ475651.1) | 95.00% | 99.47% |
| *Nairoviridae* | *Orthonairovirus* | JANV | 858 | Hunchun | Ji'an nariovirus strain NE-JA segment L(ON408088.1 ) | 98.00% | 99.52% |
| *Nairoviridae* | *Orthonairovirus* | JANV | 1,113 | Hunchun | Ji'an nairovirus isolate NE-SDG2 segment L (PQ475647.1) | 98.00% | 99.18% |
| *Nairoviridae* | *Orthonairovirus* | XCV | 639 | Hunchun | Xue-Cheng virus isolate TIGMIC 1 segment M (PP944368.1) | 97.00% | 99.03% |
| *Nairoviridae* | *Orthonairovirus* | XCV | 739 | Hunchun | Xue-Cheng virus isolate MDJ138 segment L (PP944361.1) | 100.00% | 96.88% |
| *Phenuiviridae* | *Uukuvirus* | DBTV | 426 | Hunchun | Dabieshan tick virus strain DTV/LTBMH4 segment S (MW721896.1) | 98.00% | 99.05% |
| *Phenuiviridae* | *Uukuvirus* | DBTV | 1,260 | Hunchun | Dabieshan Tick Virus isolate TIGMIC_48 (ON812317.1) | 100.00% | 99.05% |
| *Phenuiviridae* | *Uukuvirus* | DBTV | 1,320 | Hunchun | Dabieshan Tick Virus isolate TIGMIC_37 (ON812293.1) | 99.00% | 99.31% |
| *Phenuiviridae* | *Uukuvirus* | DBTV | 1,781 | Hunchun | Dabieshan tick virus isolate SDtickDTV-2018-01 segment S (MT413431.1 ) | 100.00% | 98.88% |
| *Phenuiviridae* | *Uukuvirus* | DBTV | 6,518 | Hunchun | Dabieshan tick virus isolate SDtickDTV-2018-01 segment L (MT413430.1 ) | 100.00% | 98.80% |
| *Phenuiviridae* | *Ixovirus* | STPV | 423 | Hunchun | Sara tick phlebovirus strain Rus/Ix_persulcatus/Karelia/4/2018 (MN542367.1) | 91.00% | 92.75% |
| *Phenuiviridae* | *Ixovirus* | STPV | 576 | Antu | Sara tick phlebovirus strain NE-TH4 segment L (ON408140.1) | 100.00% | 99.31% |
| *Phenuiviridae* | *Ixovirus* | STPV | 924 | Hunchun、Antu | Sara tick phlebovirus isolate STPV_BSQG1730 (OR148850.1) | 100.00% | 99.78% |
| *Phenuiviridae* | *Phlebovirus* | MKWV | 606 | Longjing | Mukawa virus strain NAC-Org5 segment M (PP999501.1 ) | 100.00% | 98.18% |
| *Phenuiviridae* | *Phlebovirus* | MKWV | 645 | Longjing | Mukawa virus strain NE-FZ2 segment M (ON408121.1 ) | 100.00% | 98.29% |
| *Phenuiviridae* | *Phlebovirus* | MKWV | 860 | Longjing | Mukawa virus segment L (MZ532501.1 ) | 100.00% | 98.72% |
| *Chuviridae* | *Mivirus* | LMV | 591 | Helong | Lesnoe mivirus isolate TIGMIC_1 (OP628497.1) | 97.00% | 100.00% |
| *Chuviridae* | *Mivirus* | LMV | 1,152 | Helong、Hunchun | Lesnoe mivirus isolate TIGMIC_14 (OP628507.1) | 100.00% | 98.87% |
| *Chuviridae* | *Mivirus* | LMV | 1,278 | Hunchun | Lesnoe mivirus isolate TIGMIC_14 (OP628507.1) | 100.00% | 98.75% |
| *Chuviridae* | *Mivirus* | LMV | 2,001 | Helong、Hunchun、Antu、Longjing | Lesnoe mivirus isolate TIGMIC_14 (OP628507.1) | 100.00% | 99.05% |
| *Chuviridae* | *Mivirus* | LMV | 5,172 | Helong、Hunchun、Antu | Lesnoe mivirus isolate TIGMIC_14 (OP628507.1) | 100.00% | 98.82% |
| *Rhabdoviridae* | *Alphanemrhavirus-like* | THRV1 | 732 | Antu | Tahe rhabdovirus 1 strain NE-DH1 (ON408166.1) | 99.00% | 99.37% |
| *Rhabdoviridae* | *Alphanemrhavirus-like* | THRV1 | 792 | Antu | Tahe rhabdovirus 1 strain NE-DH1 (ON408166.1) | 98.00% | 99.61% |
| *Rhabdoviridae* | *Alphanemrhavirus-like* | THRV1 | 873 | Antu | Tahe rhabdovirus 1 strain NE-ShL3 ( ON408168.1 ) | 100.00% | 99.43% |
| *Rhabdoviridae* | *unclassified* | YBRV4 | 627 | Antu | Yanbian Rhabd tick virus 4 isolate TIGMIC 5 (ON746526.1) | 100.00% | 99.04% |
| *Rhabdoviridae* | *unclassified* | YBRV4 | 654 | Antu | Yanbian Rhabd tick virus 4 isolate TIGMIC 5 (ON746526.1) | 99.00% | 99.54% |
| *Rhabdoviridae* | *unclassified* | YBRV4 | 655 | Antu | Yanbian Rhabd tick virus 4 isolate TIGMIC 5 (ON746526.1) | 99.00% | 99.54% |
| *Rhabdoviridae* | *unclassified* | YBRV1 | 669 | Antu | Yanbian Rhabd tick virus 1 isolate TIGMIC 4 (ON746520.1) | 100.00% | 99.70% |
| *Rhabdoviridae* | *unclassified* | YBRV1 | 1,167 | Antu | Yanbian Rhabd tick virus 1 isolate TIGMIC 4 (ON746520.1) | 100.00% | 99.74% |
| *Rhabdoviridae* | *unclassified* | MLV | 333 | Antu | Manly virus isolate HLJ-HC-3 (OP863293.1) | 94.00% | 99.68% |
| *Tombusviridae* | *Luteovirus* | CLTV3 | 717 | Hunchun | Cheeloo tick virus 3 isolate CLCM-072 (OR148393.1) | 98.00% | 96.19% |
| *Tombusviridae* | *Luteovirus* | CLTV3 | 867 | Hunchun | Cheeloo tick virus 3 isolate Hebei_094 segment 1 (PQ180797.1) | 100.00% | 99.31% |
| *Tombusviridae* | *Luteovirus* | CLTV3 | 1,050 | Hunchun | Cheeloo tick virus 3 isolate Hebei_030 segment 1 (PQ180796.1) | 100.00% | 98.86% |
| *Tombusviridae* | *Luteovirus* | NXLV | 855 | Longjing | Ningxia luteovirus strain China-NX143 (PP945028.1) | 98.00% | 98.09% |
| *Tombusviridae* | *Luteovirus* | NXLV | 1,008 | Longjing | Ningxia luteovirus strain China-NX133 (PP945029.1) | 98.00% | 97.78% |
| *Partitiviridae* | *Deltapartitivirus-like* | JLPV1 | 627 | Hunchun、Antu | Jilin partiti-like virus 1 isolate JL/QG-2(MT316409.1 ) | 100.00% | 99.68% |
| *Solemoviridae* | *Sobemo-like* | XTAV1 | 504 | Antu | Xinjiang tick associated virus 1 isolate Hebei_037 segment 2 (PQ150511.1) | 99.00% | 98.59% |
| *Solemoviridae* | *Sobemo-like* | XTAV1 | 705 | Hunchun、Antu | Xinjiang tick associated virus 1 strain DH2 (ON408202.1) | 97.00% | 99.42% |
| *Solemoviridae* | *Sobemo-like* | XTAV1 | 1,353 | Hunchun、Antu、Longjing | Xinjiang tick associated virus 1 strain DH2 (ON408202.1) | 100.00% | 99.33% |
| *Solemoviridae* | *Sobemo-like* | ISAV1 | 450 | Hunchun | Ixodes scapularis associated virus 1 strain YC4 (ON408200.1) | 92.00% | 98.79% |
| *Solemoviridae* | *Sobemo-like* | HSLV15 | 1,059 | Longjing | Hubei sobemo-like virus 15 strain China-NX143 (PP945019.1 ) | 100.00% | 97.07% |
| *Solemoviridae* | *Sobemo-like* | HSLV15 | 1,155 | Hunchun | Hubei sobemo-like virus 15 isolate CLCM-022 (OR114817.1 ) | 100.00% | 98.01% |
| *Solemoviridae* | *Sobemo-like* | HSLV15 | 1,284 | Hunchun、Longjing | Hubei sobemo-like virus 15 isolate Hebei_088 segment 2 (PQ180865.1 ) | 100.00% | 97.82% |
| *Solemoviridae* | *Sobemo-like* | HSLV15 | 1,410 | Longjing | Hubei sobemo-like virus 15 strain China-NX143 (PP945019.1 ) | 100.00% | 98.01% |
| *Solemoviridae* | *Sobemo-like* | HSLV15 | 1,614 | Hunchun | Hubei sobemo-like virus 15 isolate CLCM-092 (OR114825.1) | 100.00% | 98.70% |
| *Hepeviridae* | *unclassified Hepeviridae* | Hepelivirales sp. | 708 | Hunchun | Hepelivirales sp. isolate Hebei_027 (PQ180722.1) | 100.00% | 98.73% |
| *Hepeviridae* | *unclassified Hepeviridae* | Hepelivirales sp. | 1,059 | Hunchun | Hepelivirales sp. isolate CLCM-072(OR114691.1) | 98.00% | 96.14% |
| *Hepeviridae* | *unclassified Hepeviridae* | Hepelivirales sp. | 1,635 | Hunchun | Hepelivirales sp. isolate Hebei_027 (PQ180722.1) | 98.00% | 96.21% |
| *Hepeviridae* | *unclassified Hepeviridae* | Hepelivirales sp. | 1,896 | Hunchun | Hepelivirales sp. isolate Hebei_027 (PQ180722.1) | 100.00% | 98.21% |
| *Unclassified* | *Unclassified* | HNTV | 1,203 | Hunchun | Henan tick virus isolate CLCM-060 segment S (OR114938.1 ) | 100.00% | 95.84% |
| *Unclassified* | *Unclassified* | HNTV | 450 | Hunchun | Henan tick virus isolate CLCM-097 segment L (OR114959.1) | 96.00% | 94.71% |
| *Unclassified* | *Unclassified* | HNTV | 549 | Hunchun | Henan tick virus isolate SZSX (OR573900.1) | 97.00% | 96.25% |
| *Unclassified* | *Unclassified* | HNTV | 726 | Hunchun | Henan tick virus isolate TIGMIC_12 (ON811885.1) | 100.00% | 94.21% |
