# Supplementary material for: Metatranscriptomic Analysis Uncovers RNA Virus Diversity in Ticks From the China–Russia–North Korea Border Region
Source: Transbound Emerg Dis. 2025 Oct 12;2025:7807512. doi: 10.1155/tbed/7807512 (PMC12535811; doi:10.1155/tbed/7807512)
Supplement: Supporting Information 5 — Agarose gel electrophoresis of RNA viruses in this study. [file 7807512.f5.docx]

**Supporting Information 5.Agarose gel electrophoresis of RNA viruses in this study**

**1.Songling virus**


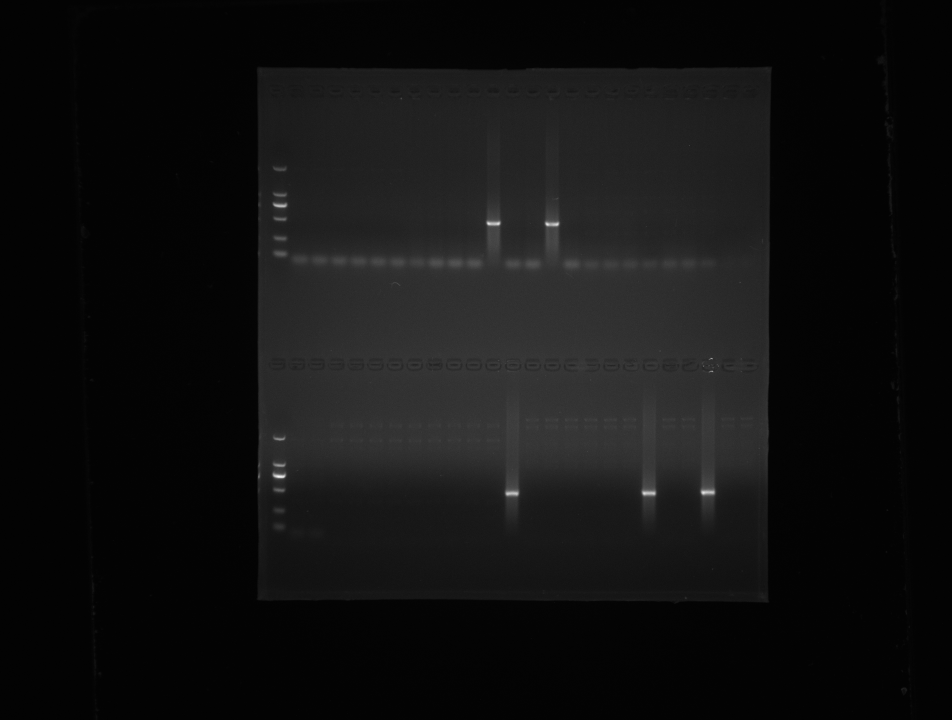

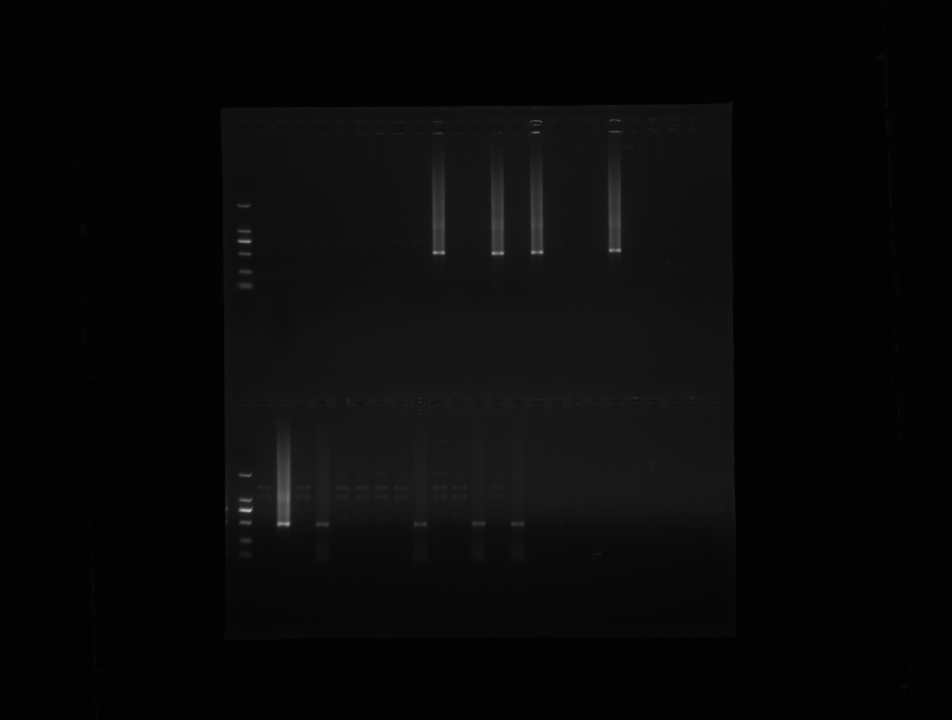


10

8

9

4

5

6

7

2

3

1

M

12

13

14

15

16

17

18

11

19

20

21

22

23

N

M 1 2 3 4 5 6 7 8 9 10 11 12 13 14 15 16 17 18 19 20 21 22 23 N

2,000 bp

1,000 bp

750 bp

500 bp

250 bp

100 bp

2,000 bp

1,000 bp

750 bp

500 bp

250 bp

100 bp

2,000 bp

1,000 bp

750 bp

500 bp

250 bp

100 bp

2,000 bp

1,000 bp

750 bp

500 bp

250 bp

100 bp


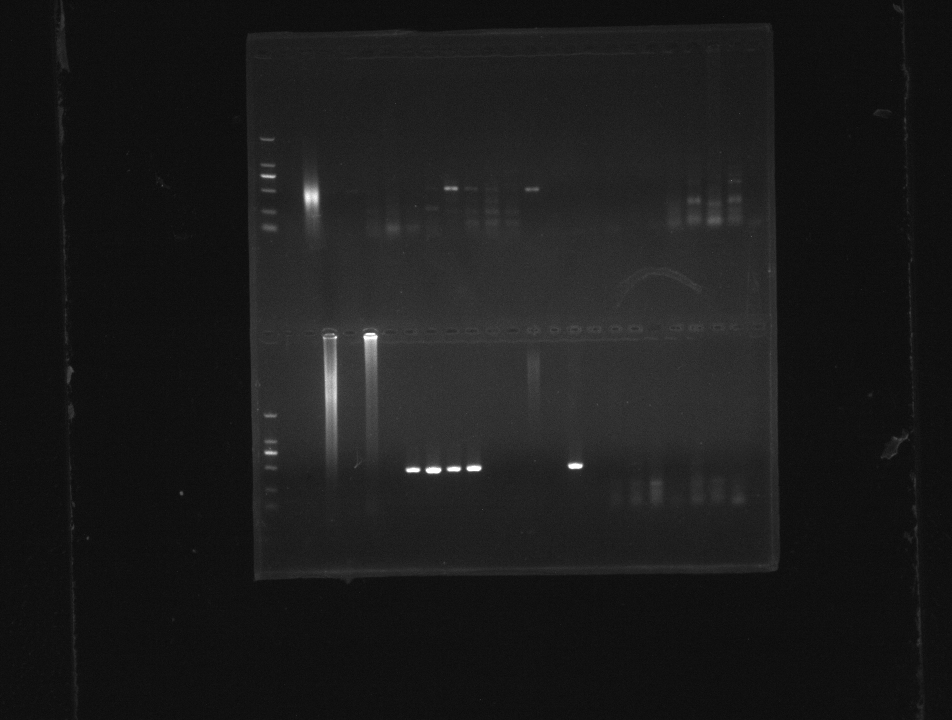


M 1 2 3 4 5 6 7 8 9 10 11 12 13 14 15 16 17 18 19 20 21 22 23 N

2,000 bp

1,000 bp

750 bp

500 bp

250 bp

100 bp

2,000 bp

1,000 bp

750 bp

500 bp

250 bp

100 bp


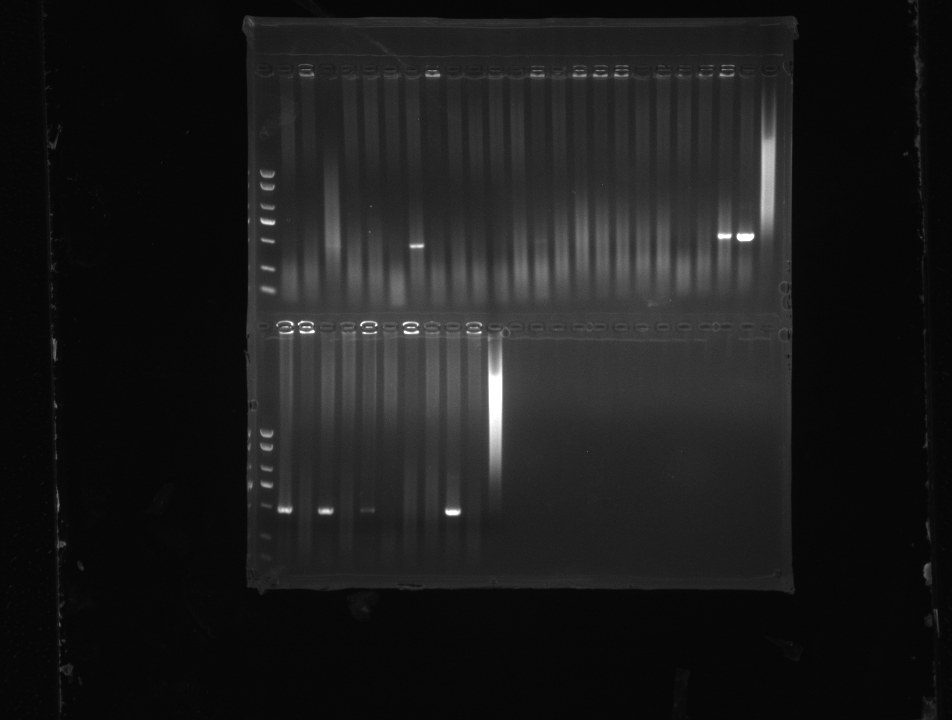


M 1 2 3 4 5 6 7 8 9 10 11 12 13 14 15 16 17 18 19 20 21 22 23 N

1,500 bp

100 bp

250 bp

500 bp

750 bp

1,000 bp

2,000 bp

1,500 bp

1,000 bp

750 bp

500 bp

250 bp

100 bp

2,000 bp


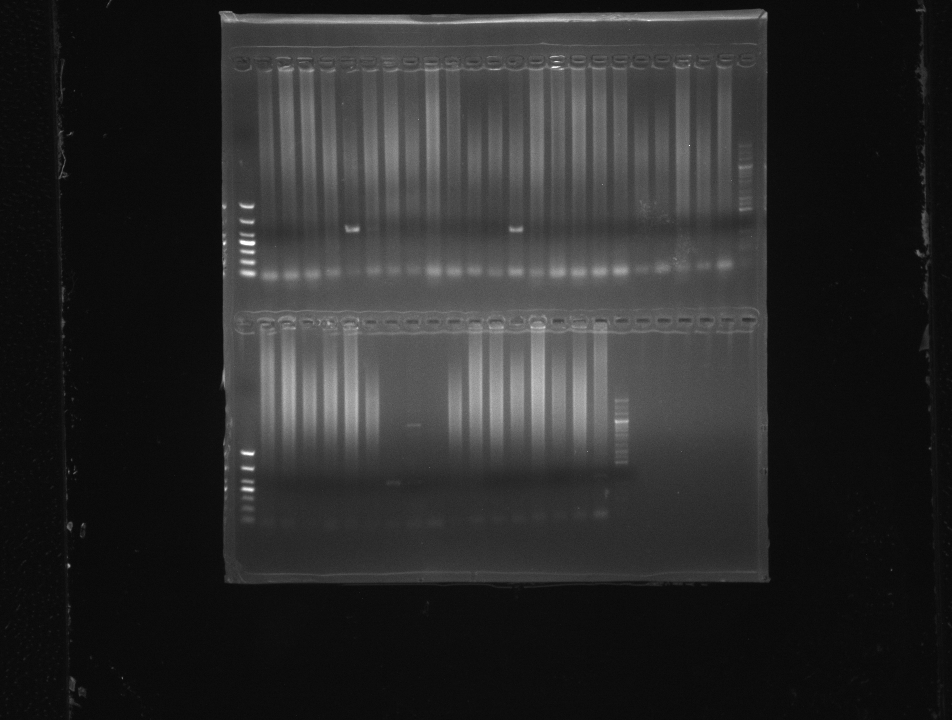


700 bp

700 bp

M 1 2 3 4 5 6 7 8 9 10 11 12 13 14 15 16 17 18 19 20 21 22 23 N

1,000 bp

500 bp

400 bp

300 bp

200 bp

100 bp

1,000 bp

500 bp

400 bp

300 bp

200 bp

100 bp

**2.Ji'an nairovirus**


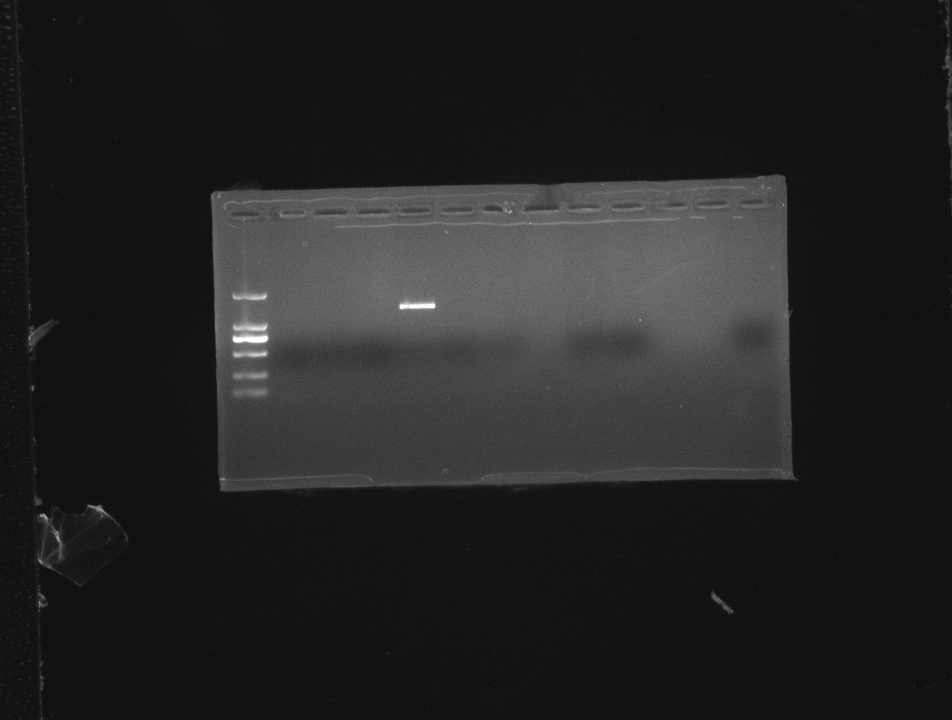


M 1 2 3 4 5 6 7 8 9 10 11 N

2,000 bp

1,000 bp

750 bp

500 bp

250 bp

100 bp


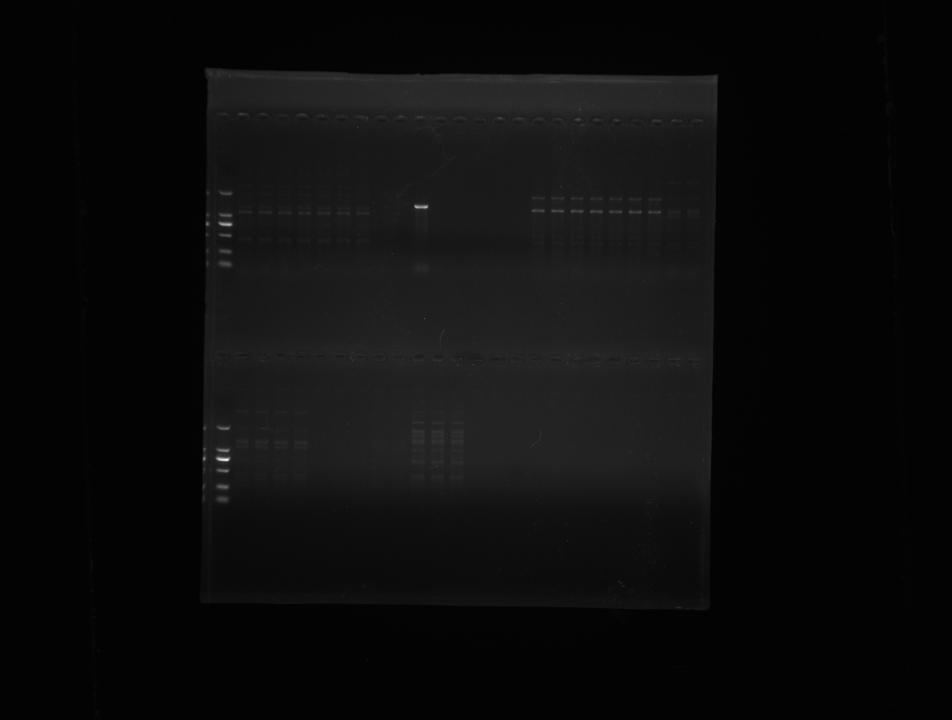


M 1 2 3 4 5 6 7 8 9 10 11 12 13 14 15 16 17 18 19 20 21 22 23 N

2,000 bp

1,000 bp

750 bp

500 bp

250 bp

100 bp

2,000 bp

1,000 bp

750 bp

500 bp

250 bp

100 bp

**3.Dabieshan tick virus**


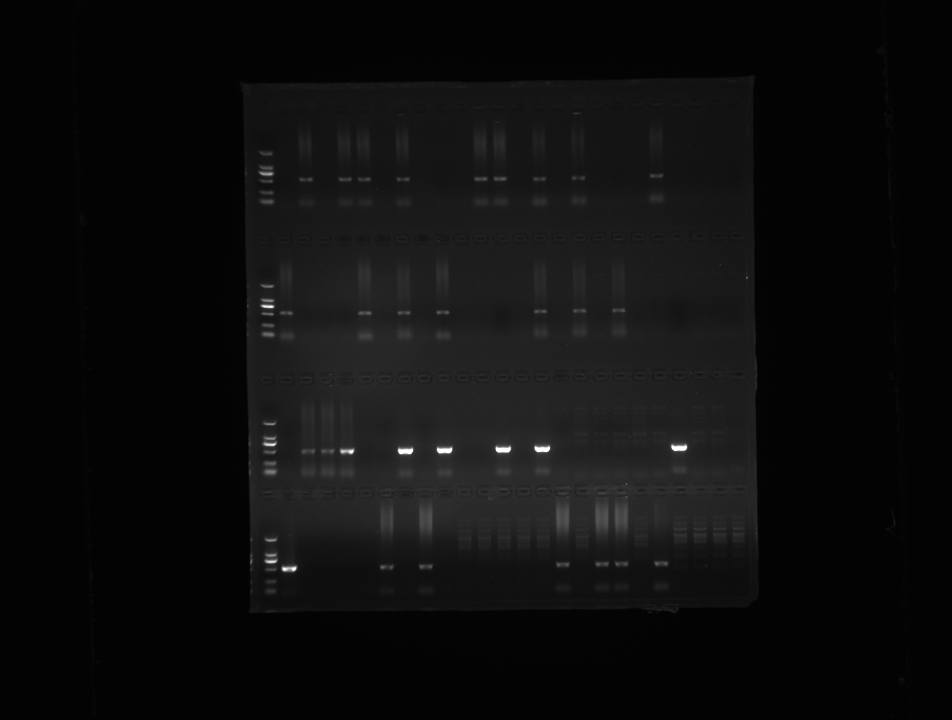


M 1 2 3 4 5 6 7 8 9 10 11 12 13 14 15 16 17 18 19 20 21 22 23 N

2,000 bp

1,000 bp

750 bp

500 bp

250 bp

100 bp

2,000 bp

1,000 bp

750 bp

500 bp

250 bp

100 bp

2,000 bp

1,000 bp

750 bp

500 bp

250 bp

100 bp

2,000 bp

1,000 bp

750 bp

500 bp

250 bp

100 bp

**4.Hubei sobemo-like virus 15**


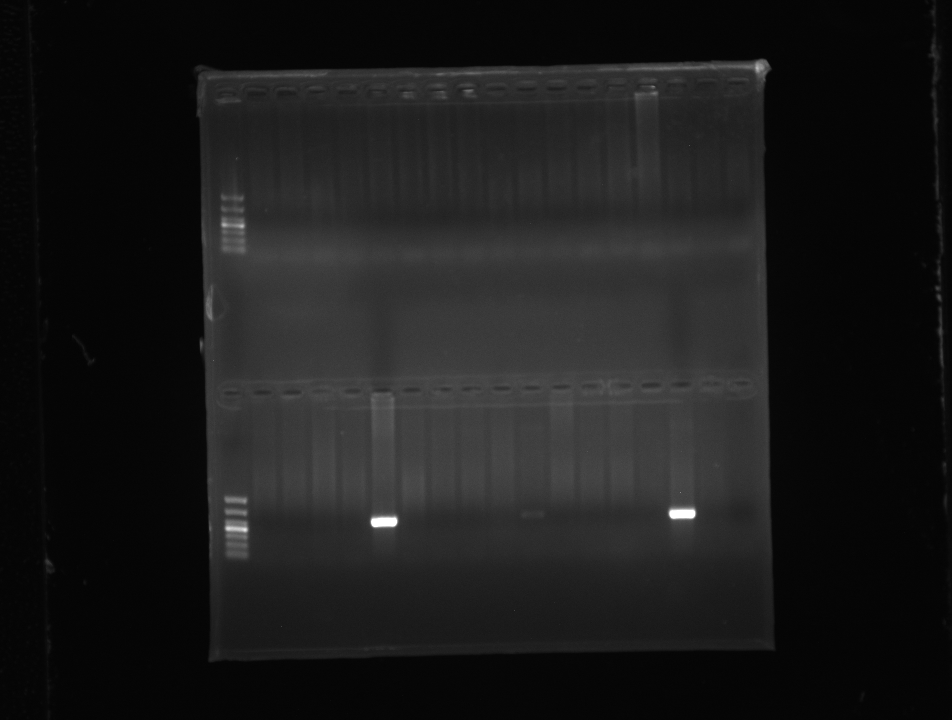


M 1 2 3 4 5 6 7 8 9 10 11 12 13 14 15 16 N

1,500 bp

2,000 bp

1,000 bp

750 bp

500 bp

250 bp

100 bp

1,500 bp

2,000 bp

1,000 bp

750 bp

500 bp

250 bp

100 bp


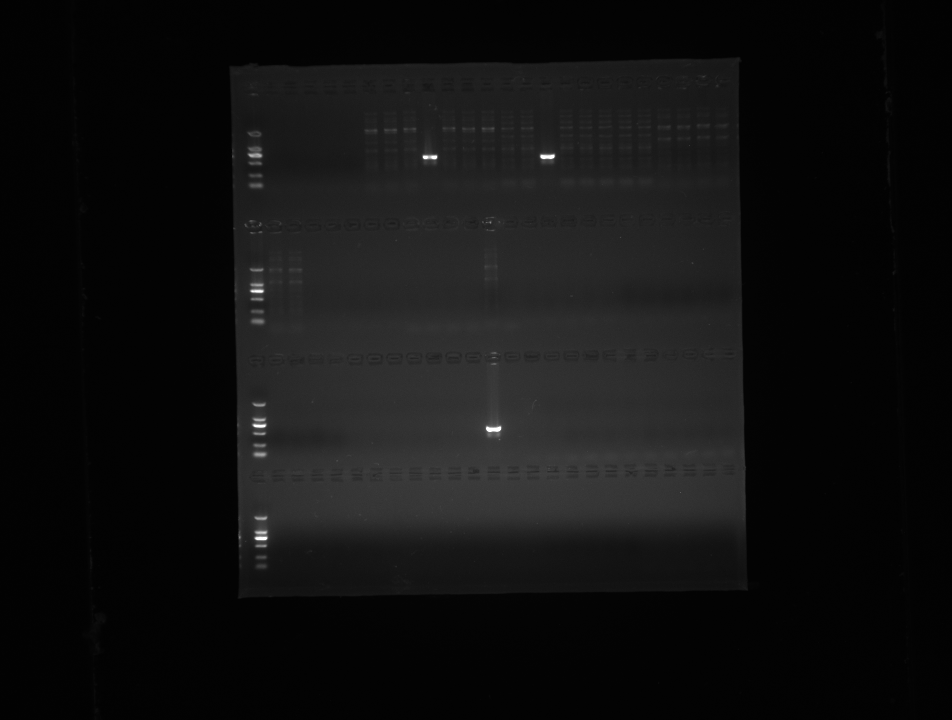


M 1 2 3 4 5 6 7 8 9 10 11 12 13 14 15 16 17 18 19 20 21 22 23 N

2,000 bp

1,000 bp

750 bp

500 bp

250 bp

100 bp

2,000 bp

1,000 bp

750 bp

500 bp

250 bp

100 bp

2,000 bp

1,000 bp

750 bp

500 bp

250 bp

100 bp

2,000 bp

1,000 bp

750 bp

500 bp

250 bp

100 bp

**5.Xue-Cheng virus**


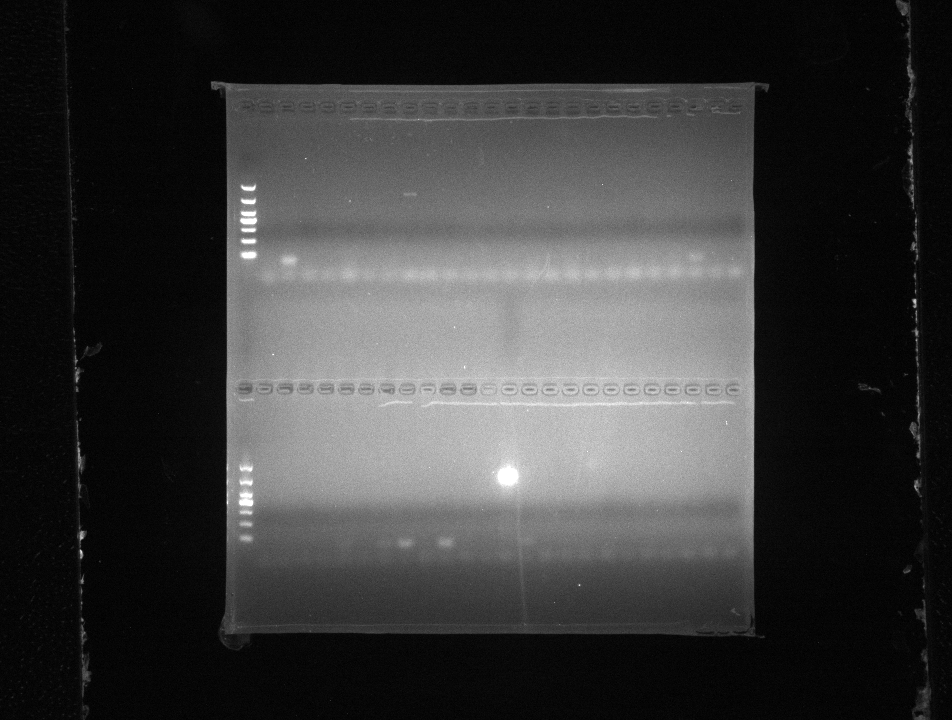

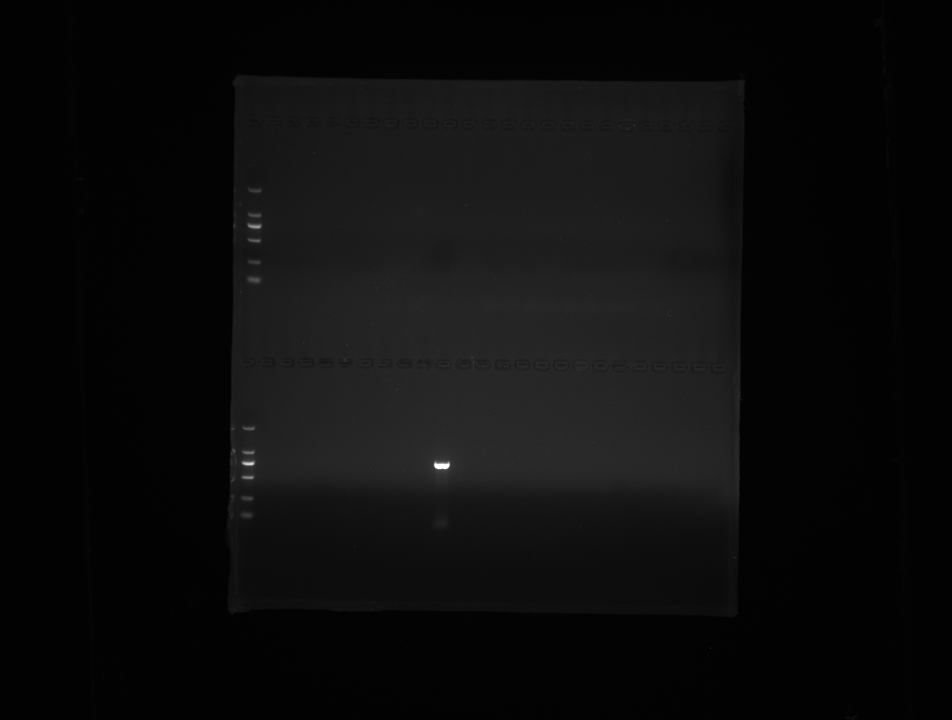


700 bp

1,000 bp

500 bp

400 bp

300 bp

200 bp

100 bp

700 bp

M 1 2 3 4 5 6 7 8 9 10 11 12 13 14 15 16 17 18 19 20 21 22 23 N

M 1 2 3 4 5 6 7 8 9 10 11 12 13 14 15 16 17 18 19 20 21 22 23 N

2,000 bp

1,000 bp

750 bp

500 bp

250 bp

100 bp

2,000 bp

1,000 bp

750 bp

500 bp

250 bp

100 bp

1,000 bp

500 bp

400 bp

300 bp

200 bp

100 bp

**6.Cheeloo tick virus 3**


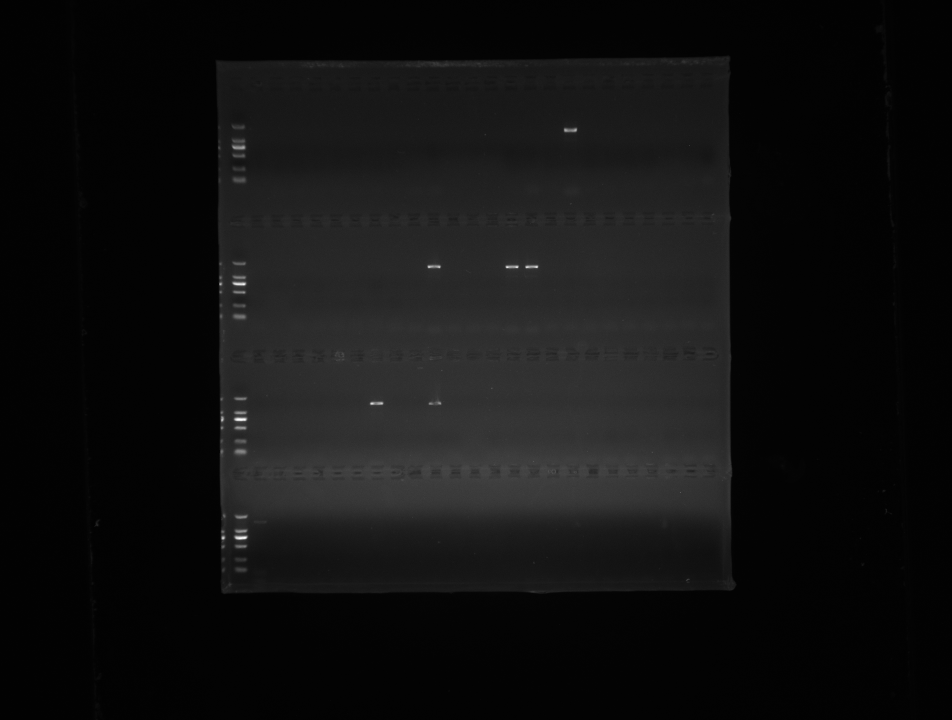


M 1 2 3 4 5 6 7 8 9 10 11 12 13 14 15 16 17 18 19 20 21 22 23 N

2,000 bp

1,000 bp

750 bp

500 bp

250 bp

100 bp

2,000 bp

1,000 bp

750 bp

500 bp

250 bp

100 bp

2,000 bp

1,000 bp

750 bp

500 bp

250 bp

100 bp

2,000 bp

1,000 bp

750 bp

500 bp

250 bp

100 bp

**7.Xinjiang tick associated virus 1**


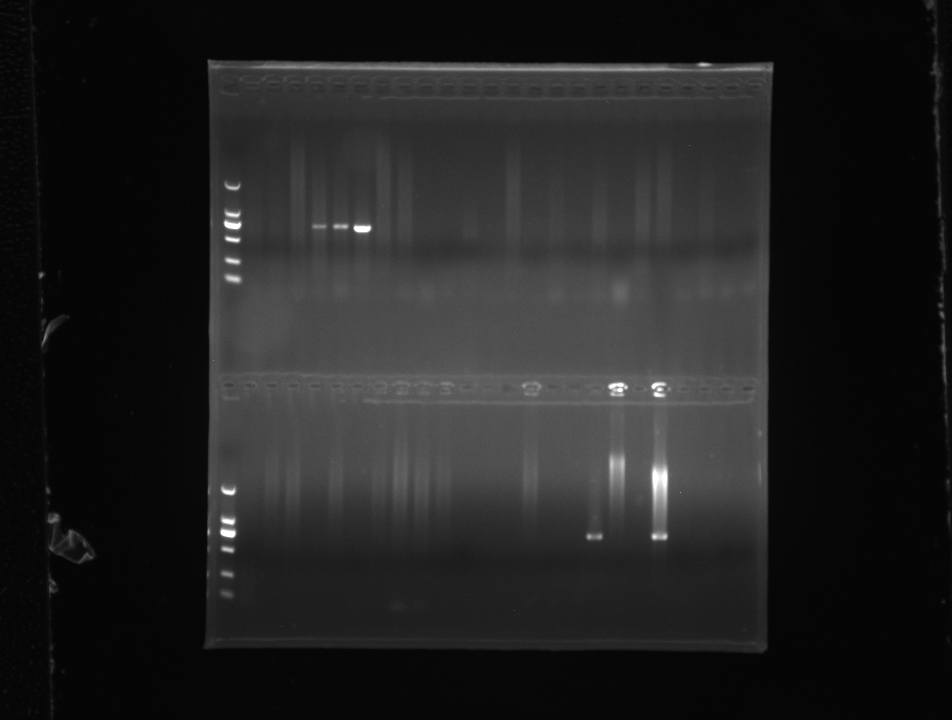


M 1 2 3 4 5 6 7 8 9 10 11 12 13 14 15 16 17 18 19 20 21 22 23 N

2,000 bp

1,000 bp

750 bp

500 bp

250 bp

100 bp

2,000 bp

1,000 bp

750 bp

500 bp

250 bp

100 bp


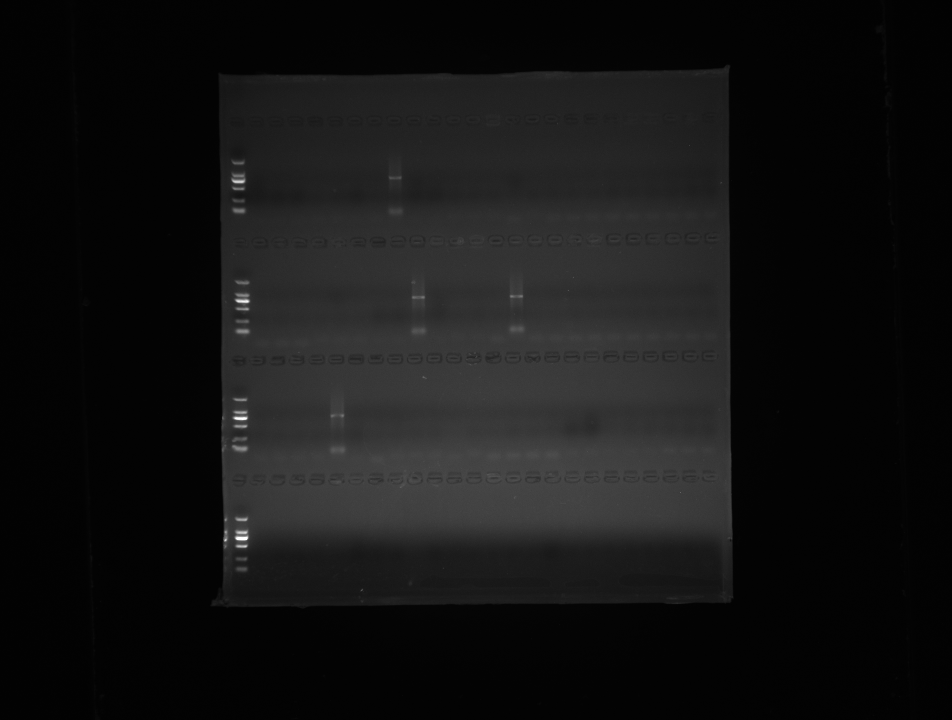


M 1 2 3 4 5 6 7 8 9 10 11 12 13 14 15 16 17 18 19 20 21 22 23 N

2,000 bp

1,000 bp

750 bp

500 bp

250 bp

100 bp

2,000 bp

1,000 bp

750 bp

500 bp

250 bp

100 bp

2,000 bp

1,000 bp

750 bp

500 bp

250 bp

100 bp

2,000 bp

1,000 bp

750 bp

500 bp

250 bp

100 bp

**8.Hepelivirales sp.**


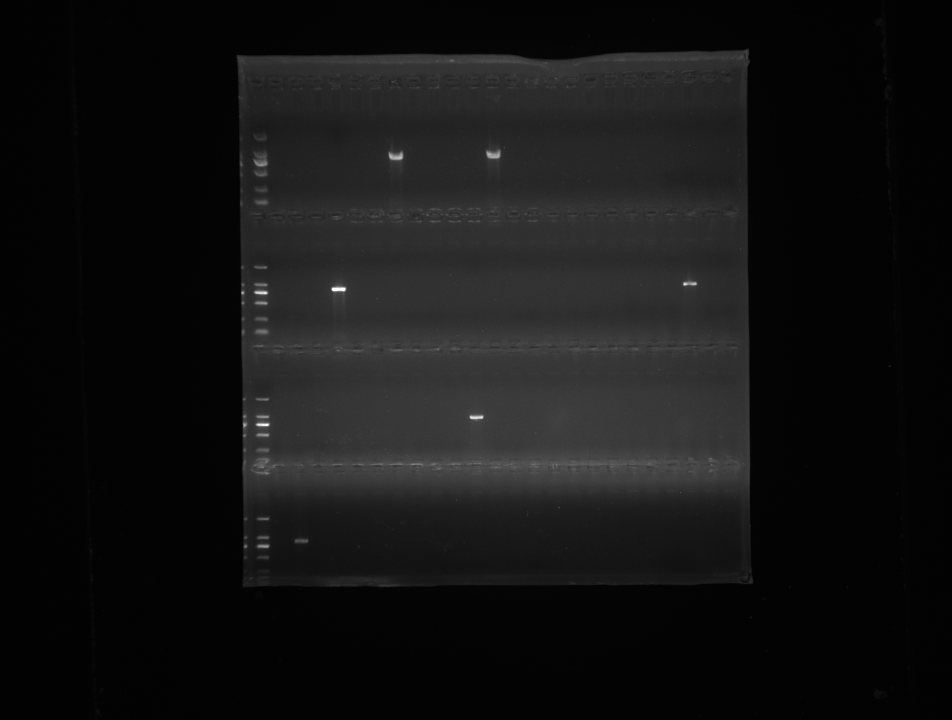


M 1 2 3 4 5 6 7 8 9 10 11 12 13 14 15 16 17 18 19 20 21 22 23 N

2,000 bp

1,000 bp

750 bp

500 bp

250 bp

100 bp

2,000 bp

1,000 bp

750 bp

500 bp

250 bp

100 bp

2,000 bp

1,000 bp

750 bp

500 bp

250 bp

100 bp

2,000 bp

1,000 bp

750 bp

500 bp

250 bp

100 bp

**9.Ixodes scapularis associated virus 1**

**
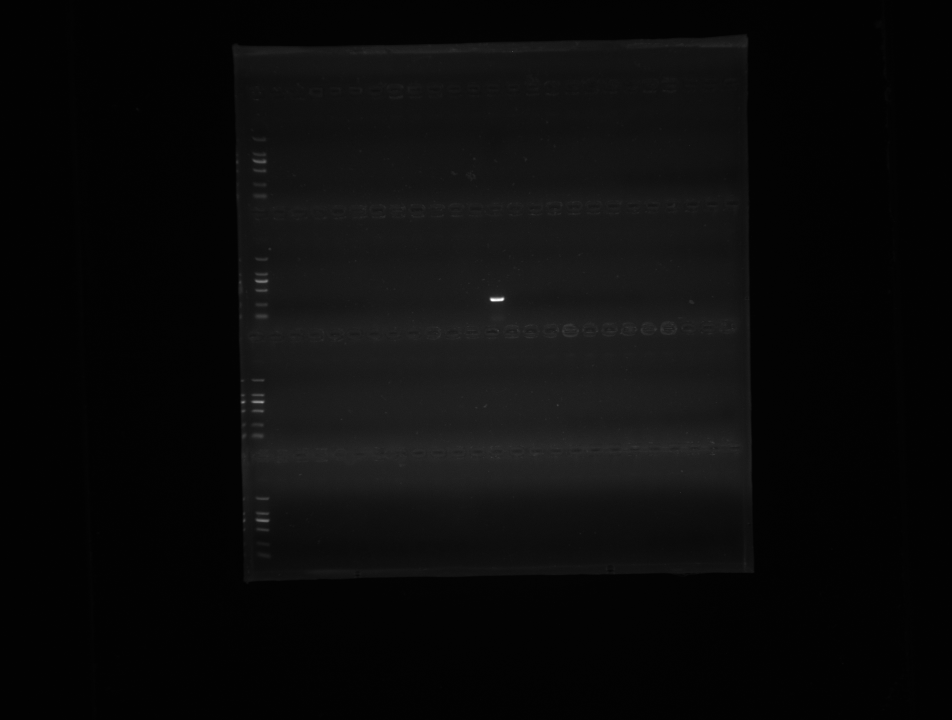
**

M 1 2 3 4 5 6 7 8 9 10 11 12 13 14 15 16 17 18 19 20 21 22 23 N

2,000 bp

1,000 bp

750 bp

500 bp

250 bp

100 bp

2,000 bp

1,000 bp

750 bp

500 bp

250 bp

100 bp

2,000 bp

1,000 bp

750 bp

500 bp

250 bp

100 bp

2,000 bp

1,000 bp

750 bp

500 bp

250 bp

100 bp

**10.Jilin partiti-like virus 1**


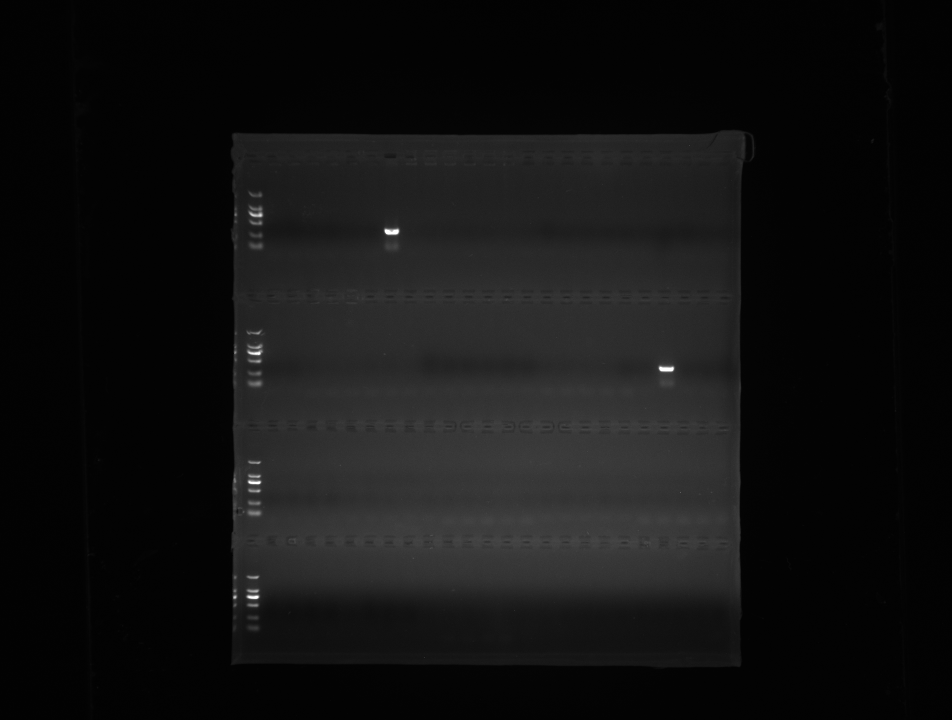


M 1 2 3 4 5 6 7 8 9 10 11 12 13 14 15 16 17 18 19 20 21 22 23 N

2,000 bp

1,000 bp

750 bp

500 bp

250 bp

100 bp

2,000 bp

1,000 bp

750 bp

500 bp

250 bp

100 bp

2,000 bp

1,000 bp

750 bp

500 bp

250 bp

100 bp

2,000 bp

1,000 bp

750 bp

500 bp

250 bp

100 bp

**11.Sara tick phlebovirus**

**
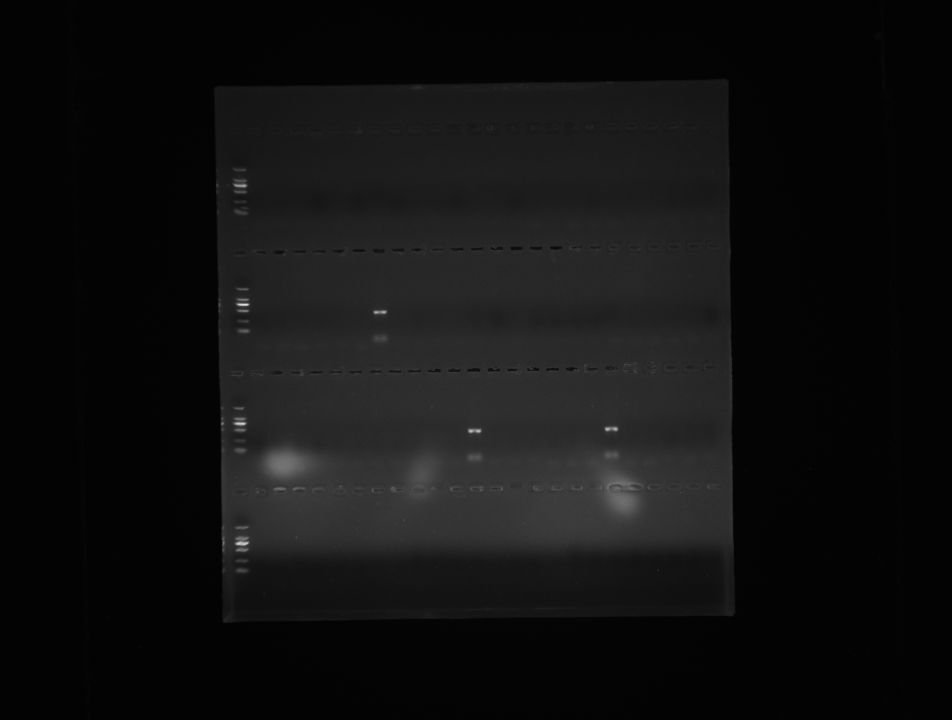
**

M 1 2 3 4 5 6 7 8 9 10 11 12 13 14 15 16 17 18 19 20 21 22 23 N

2,000 bp

1,000 bp

750 bp

500 bp

250 bp

100 bp

2,000 bp

1,000 bp

750 bp

500 bp

250 bp

100 bp

2,000 bp

1,000 bp

750 bp

500 bp

250 bp

100 bp

M 1 2 3 4 5 6 7 8 9 10 11 12 13 14 15 16 17 18 19 20 21 22 23 N

2,000 bp

1,000 bp

750 bp

500 bp

250 bp

100 bp

**12.Yanbian Rhabd tick virus 4**

**
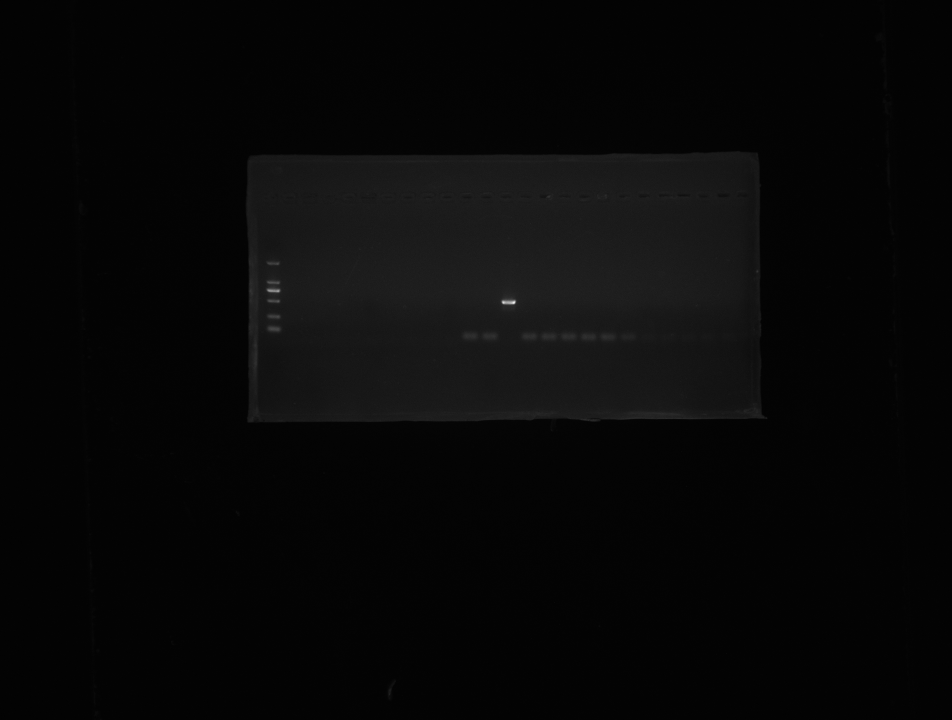
**

2,000 bp

1,000 bp

750 bp

500 bp

250 bp

100 bp

**
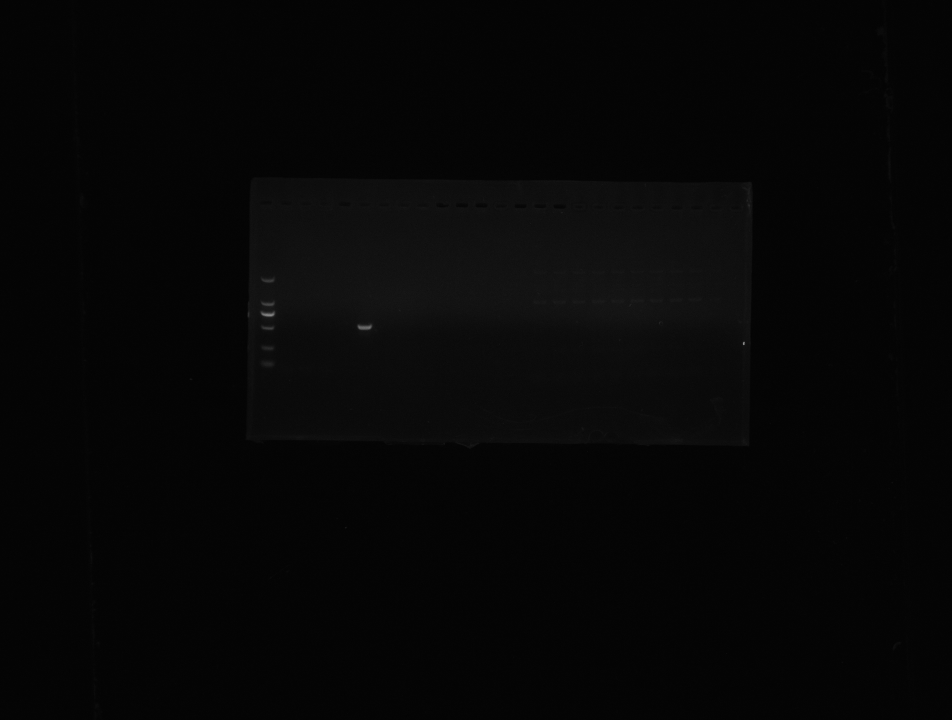
**

M 1 2 3 4 5 6 7 8 9 10 11 12 13 14 15 16 17 18 19 20 21 22 23 N

2,000 bp

1,000 bp

750 bp

500 bp

250 bp

100 bp

**13.Tahe rhabdovirus 1**

**
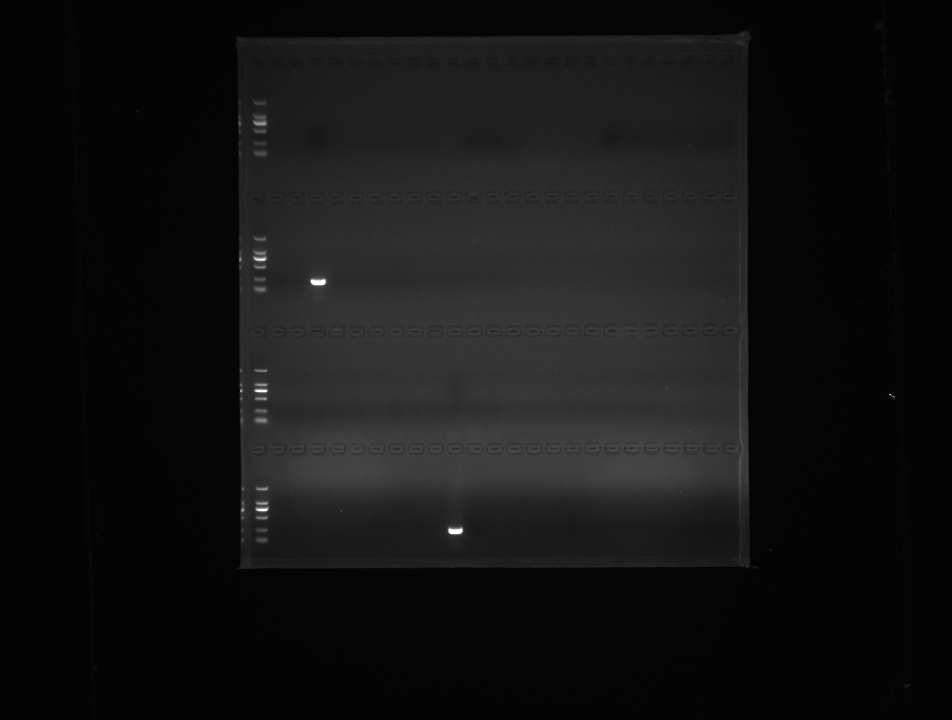
**

2,000 bp

1,000 bp

750 bp

500 bp

250 bp

100 bp

2,000 bp

1,000 bp

750 bp

500 bp

250 bp

100 bp

2,000 bp

1,000 bp

750 bp

500 bp

250 bp

100 bp

2,000 bp

1,000 bp

750 bp

500 bp

250 bp

100 bp

M 1 2 3 4 5 6 7 8 9 10 11 12 13 14 15 16 17 18 19 20 21 22 23 N

**14.Yanbian Rhabd tick virus 1**

**
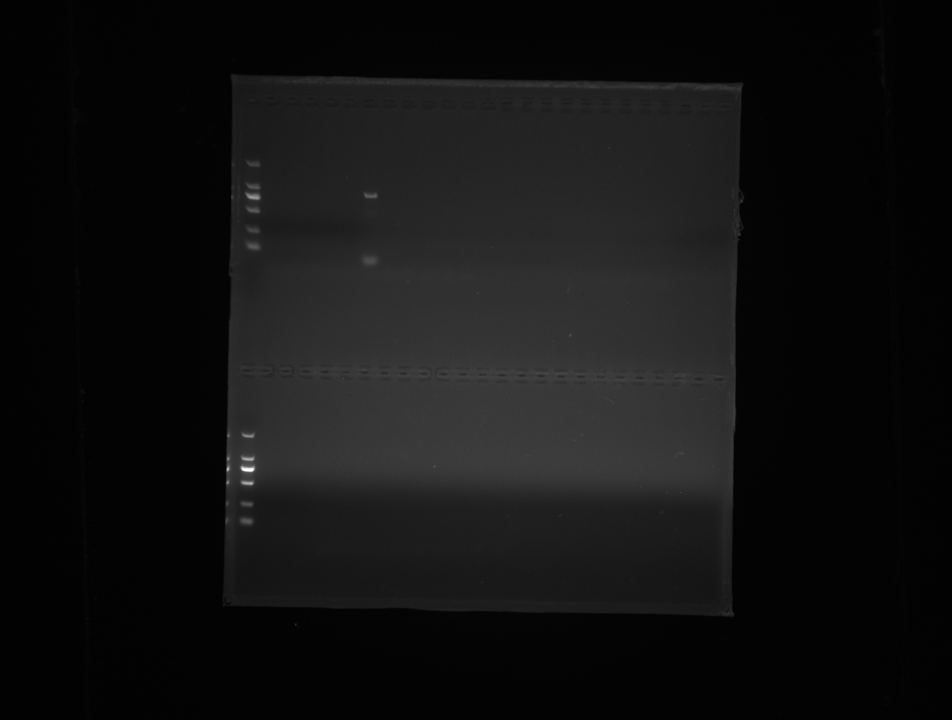
**

M 1 2 3 4 5 6 7 8 9 10 11 12 13 14 15 16 17 18 19 20 21 22 23 N

2,000 bp

1,000 bp

750 bp

500 bp

250 bp

100 bp

2,000 bp

1,000 bp

750 bp

500 bp

250 bp

100 bp

M 1 2 3 4 5 6 7 8 9 10 11 12 13 14 15 16 17 18 19 20 21 22 23 N

**15.Hunchun nairovirus**

**
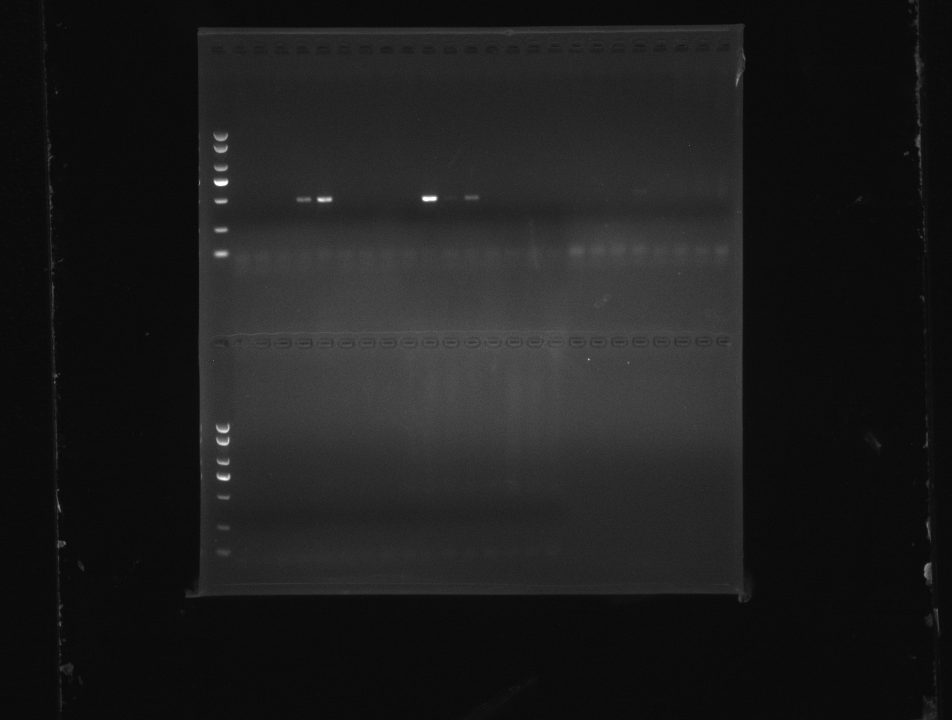
**

2,000 bp

1,000 bp

750 bp

500 bp

250 bp

100 bp

1,500 bp

2,000 bp

1,000 bp

750 bp

500 bp

250 bp

100 bp

1,500 bp

**16.Mukawa phlebovirus**

**
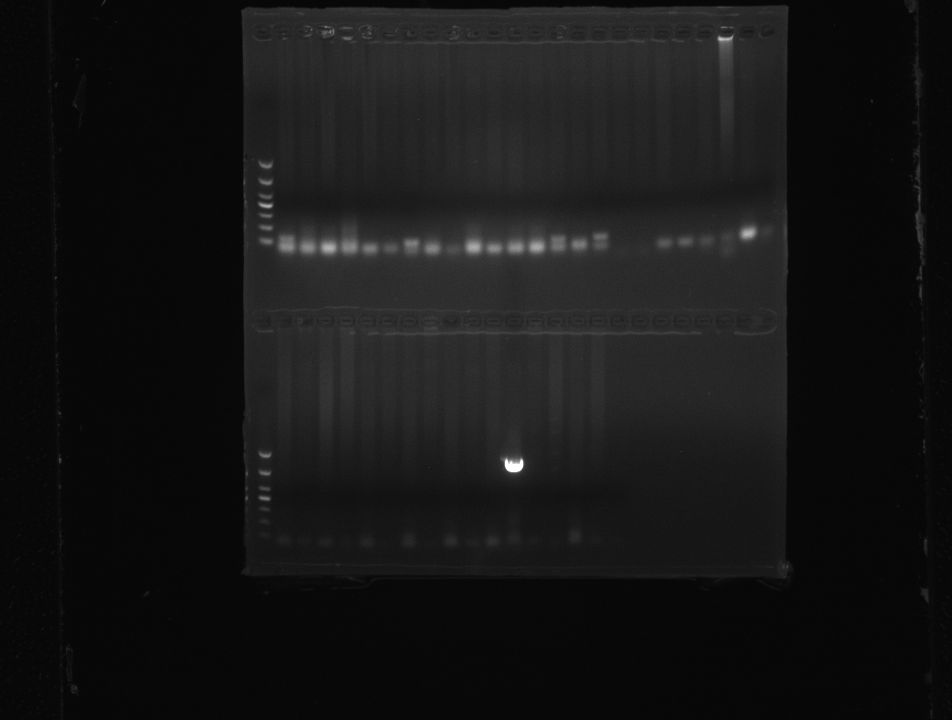
**

M 1 2 3 4 5 6 7 8 9 10 11 12 13 14 15 16 17 18 19 20 21 22 23 N

1,000 bp

500 bp

400 bp

300 bp

200 bp

100 bp

700 bp

1,000 bp

500 bp

400 bp

300 bp

200 bp

100 bp

700 bp

**17.Yanggou tick virus**

M 1 2 3 4 5 6 7 8 9 10 11 12 13 14 15 16 17 18 19 20 21 22 23 N

**
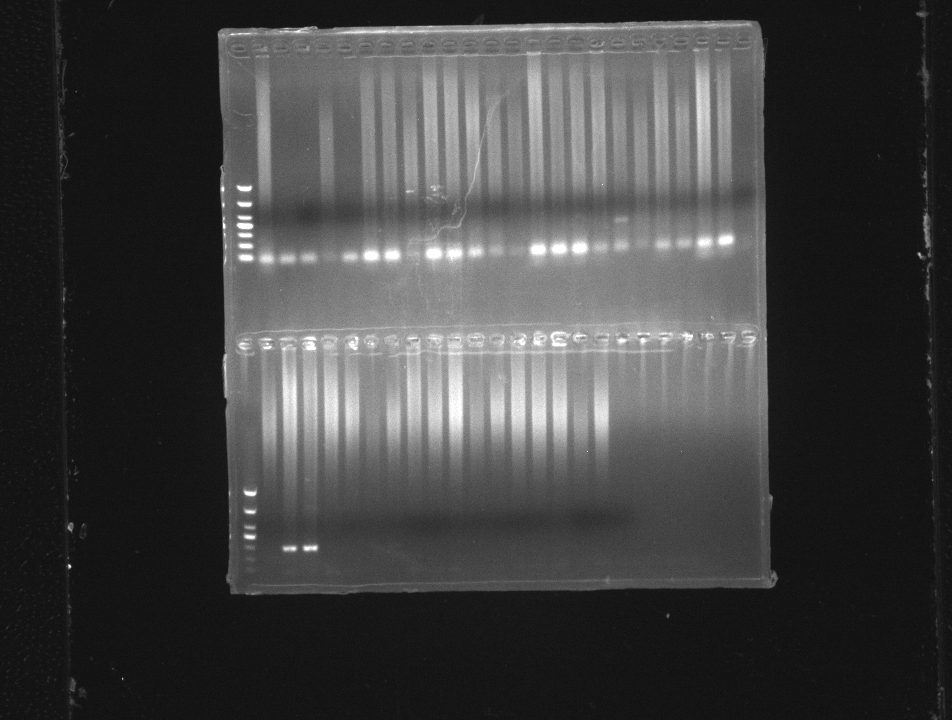
**

1,000 bp

500 bp

400 bp

300 bp

200 bp

100 bp

700 bp

1,000 bp

500 bp

400 bp

300 bp

200 bp

100 bp

700 bp

**18.Beiji nairovirus**

M 1 2 3 4 5 6 7 8 9 10 11 12 13 14 15 16 17 18 19 20 21 22 23 N

**
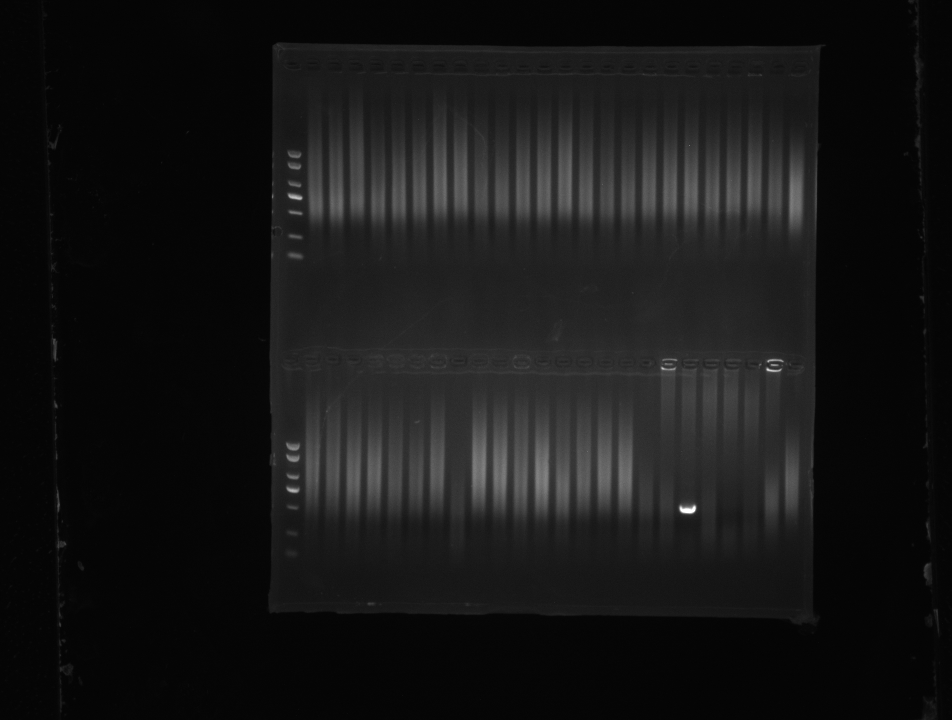
**

2,000 bp

1,000 bp

750 bp

500 bp

250 bp

100 bp

1,500 bp

2,000 bp

1,000 bp

750 bp

500 bp

250 bp

100 bp

1,500 bp

**19.Ningxia luteovirus**

M 1 2 3 4 5 6 7 8 9 10 11 12 13 14 15 16 N

**
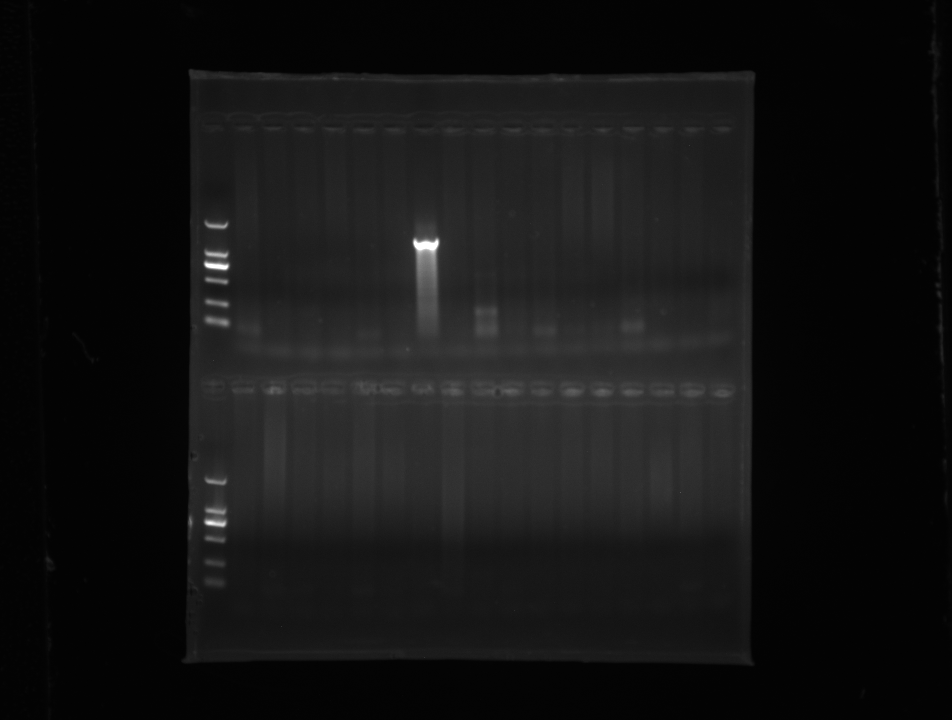
**

2,000 bp

1,000 bp

750 bp

500 bp

250 bp

100 bp

2,000 bp

1,000 bp

750 bp

500 bp

250 bp

100 bp

**20.Manly virus**

M 1 2 3 4 5 6 7 8 9 10 11 12 13 14 15 16 N

**
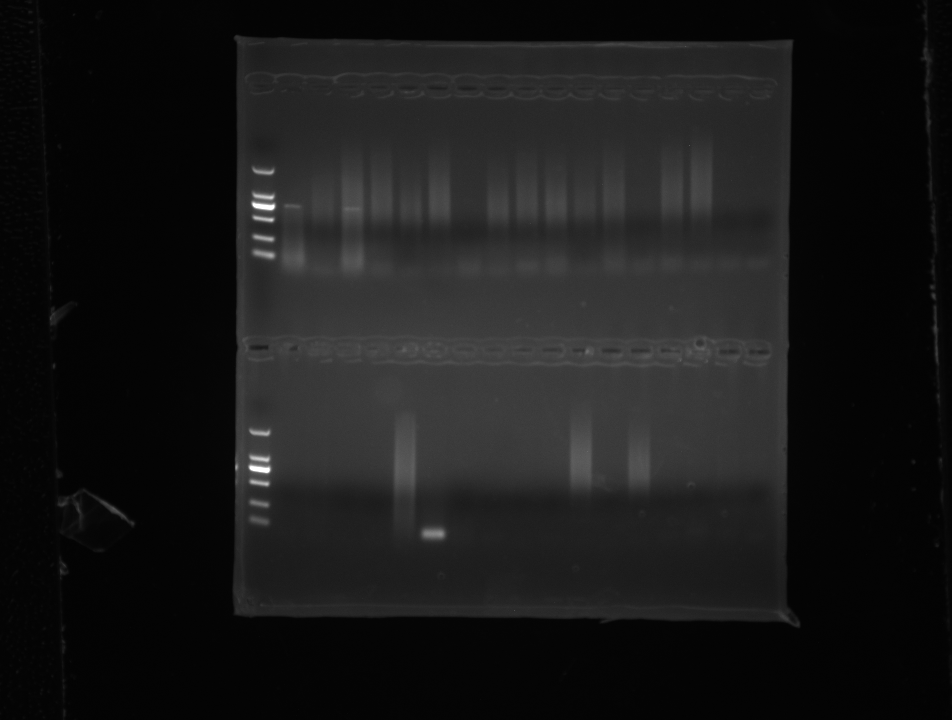
**

2,000 bp

1,000 bp

750 bp

500 bp

250 bp

100 bp

2,000 bp

1,000 bp

750 bp

500 bp

250 bp

100 bp

**21.Lesnoe mivirus**

M 1 2 3 4 5 6 7 8 9 10 11 12 13 14 15 16 N

**
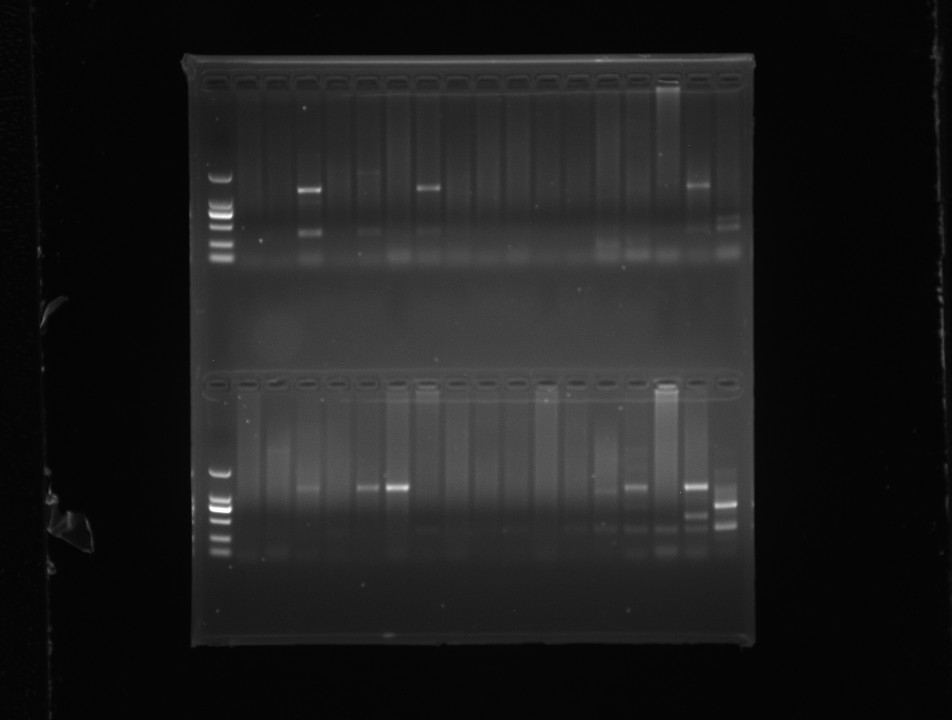
**

2,000 bp

1,000 bp

750 bp

500 bp

250 bp

100 bp

2,000 bp

1,000 bp

750 bp

500 bp

250 bp

100 bp

**
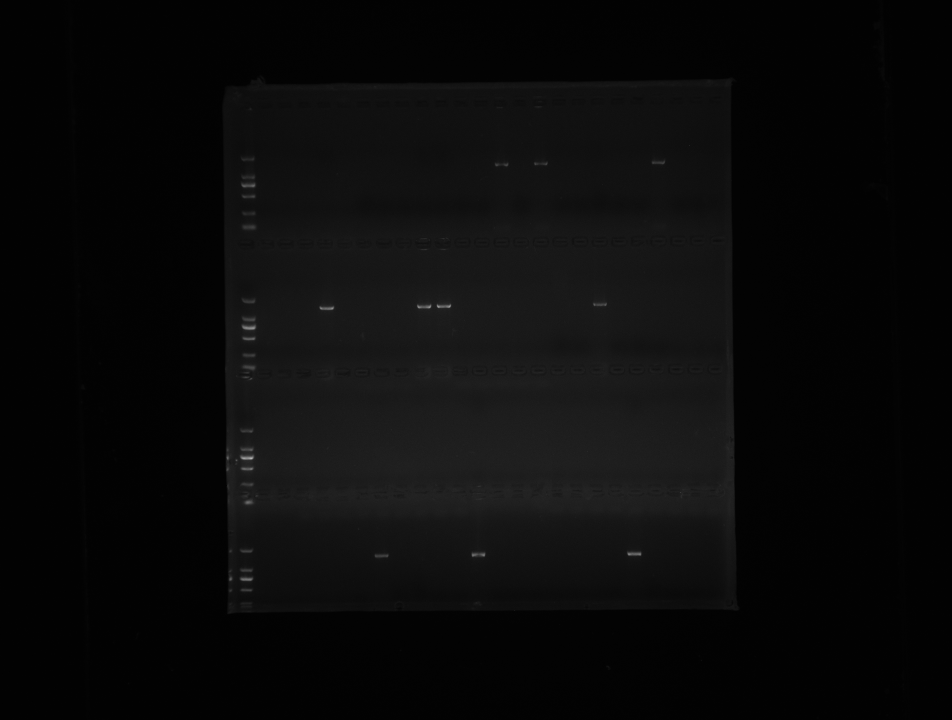
**

M 1 2 3 4 5 6 7 8 9 10 11 12 13 14 15 16 17 18 19 20 21 22 23 N

M 1 2 3 4 5 6 7 8 9 10 11 12 13 14 15 16 17 18 19 20 21 22 23 N

2,000 bp

1,000 bp

750 bp

500 bp

250 bp

100 bp

2,000 bp

1,000 bp

750 bp

500 bp

250 bp

100 bp

2,000 bp

1,000 bp

750 bp

500 bp

250 bp

100 bp

2,000 bp

1,000 bp

750 bp

500 bp

250 bp

100 bp

**
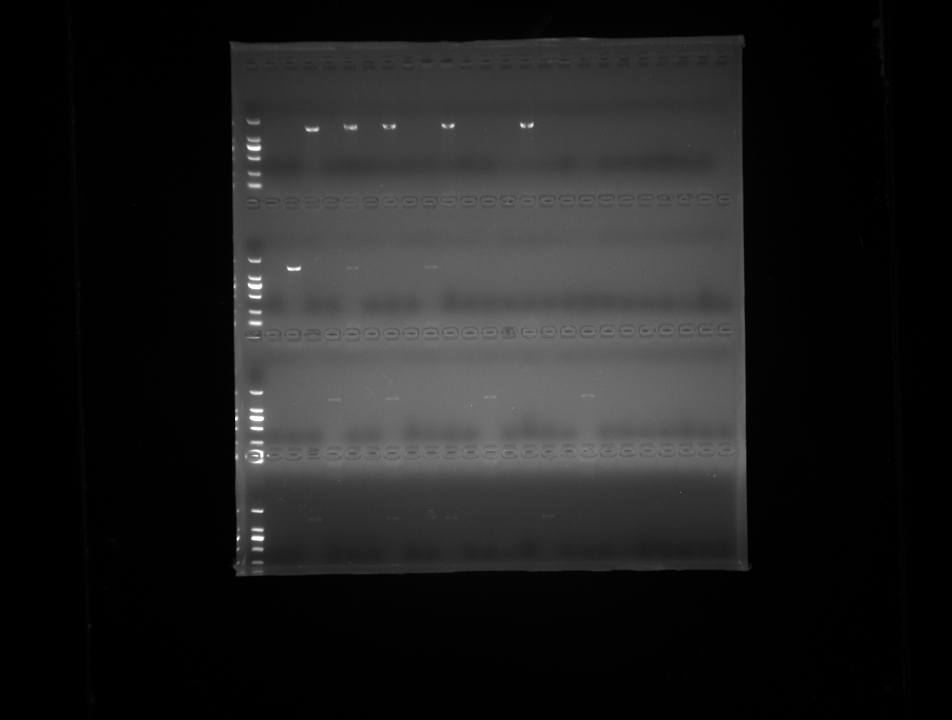
**

2,000 bp

1,000 bp

750 bp

500 bp

250 bp

100 bp

2,000 bp

1,000 bp

750 bp

500 bp

250 bp

100 bp

2,000 bp

1,000 bp

750 bp

500 bp

250 bp

100 bp

2,000 bp

1,000 bp

750 bp

500 bp

250 bp

100 bp
