# Supplementary material for: Metatranscriptomic Analysis Uncovers RNA Virus Diversity in Ticks From the China–Russia–North Korea Border Region
Source: Transbound Emerg Dis. 2025 Oct 12;2025:7807512. doi: 10.1155/tbed/7807512 (PMC12535811; doi:10.1155/tbed/7807512)
Supplement: Supporting Information 6 — PCR-based survey of tick-borne viruses infecting humans and animals in the China–Russia–North Korea border region. [file 7807512.f6.docx]

**Supporting Information 6. PCR-Based Survey of Tick-Borne Viruses Infecting Humans and Animals in the China-Russia-North Korea Border Region.**

| **Virus species** | **Total no. (%) ticks positive [95% CI]** | | | | | | | | | | | | | | | |
| --- | --- | --- | --- | --- | --- | --- | --- | --- | --- | --- | --- | --- | --- | --- | --- | --- |
|  | **Helong** | | | **Hunchun** | | | | | **Antu** | | | | **Longjing** | | | |
|  | ***H. concinna*** | ***H. japonica*** | ***I. persulcatus*** | ***H. longicornis*** | ***H. concinna*** | ***H. japonica*** | ***I. persulcatus*** | ***D. silvarum*** | ***H. concinna*** | ***H. japonica*** | ***I. persulcatus*** | ***D. silvarum*** | ***H. concinna*** | ***H. japonica*** | ***I. persulcatus*** | ***D. silvarum*** |
| Lesnoe mivirus | 9/41 (2.75) [ 1.37 - 5.00 ] | 4/6 (43.82)  [ 16.25 - 80.58 ] | 0 | 0 | 6/11 (32.65)  [ 15.2 7- 59.08 ] | 4/11 (11.43)  [ 4.11 - 25.15 ] | 0 | 0 | 0 | 1/12 (1.57)  [ 0.09 - 7.40 ] | 0 | 1/44 (0.27)  [ 0.02 - 1.29 ] | 6/27 (2.79)  [ 1.17 - 5.74 ] | 2/7 (5.15)  [ 1.02 - 16.53 ] | 0 | 1/5 (5.16)  [ 0.39 - 22.67 ] |
| Songling virus | 7/41 (2.21)  [ 0.94 - 4.18 ] | 0 | 0 | 3/59 (0.62)   [0.16 - 1.68 ] | 0 | 0 | 1/15 (1.19)  [ 0.07 - 5.83 ] | 0 | 5/17 (4.69)  [ 1.87 - 10.05 ] | 0 | 2/9 (6.44)  [ 1.44 - 18.64 ] | 0 | 14/27 (7.69)  [ 4.55 - 12.46 ] | 0 | 0 | 0 |
| Dabieshan tick virus | 0 | 0 | 0 | 31/59 (8.94)  [ 6.25 - 12.53 ] | 0 | 0 | 0 | 0 | 0 | 0 | 0 | 0 | 0 | 0 | 0 | 0 |
| Xinjiang tick associated virus 1 | 0 | 0 | 0 | 0 | 0 | 0 | 0 | 1/11 (3.22)  [ 0.18 - 16.28 ] | 0 | 0 | 0 | 6/44 (1.70)  [ 0.70 - 3.52 ] | 0 | 0 | 0 | 2/5 (17.55)  [ 3.30 - 61.98 ] |
| Cheeloo tick virus 3 | 0 | 0 | 0 | 7/59 (1.49)  [ 0.66 - 2.92 ] | 0 | 0 | 0 | 0 | 0 | 0 | 0 | 0 | 0 | 0 | 0 | 0 |
| Hubei sobemo-like virus 15 | 0 | 0 | 0 | 2/59 (0.41)  [ 0.07 - 1.34 ] | 0 | 1/11 (2.65)  [ 0.16 - 12.24 ] | 0 | 0 | 0 | 0 | 0 | 0 | 1/27 (0.43)  [ 0.02 - 2.07 ] | 1/7 (2.58)  [0.15-12.81] | 0 | 1/5 (5.16)  [ 0.39 - 22.67 ] |
| Hepelivirales sp. | 0 | 0 | 0 | 6/59 (1.27)  [ 0.53 - 2.63 ] | 0 | 0 | 0 | 0 | 0 | 0 | 0 | 0 | 0 | 0 | 0 | 0 |
| Hunchun nairovirus | 0 | 0 | 0 | 0 | 0 | 0 | 4/15 (5.00)  [ 1.76 - 11.55 ] | 0 | 0 | 0 | 0 | 0 | 0 | 0 | 0 | 0 |
| Yanbian Rhabd tick virus 1 | 0 | 0 | 0 | 0 | 0 | 0 | 0 | 0 | 0 | 0 | 1/9 (4.15)  [ 0.22 - 22.15 ] | 0 | 0 | 0 | 0 | 0 |
| Yanbian Rhabd tick virus 4 | 0 | 0 | 0 | 0 | 0 | 0 | 0 | 0 | 0 | 2/12 (3.40)  [ 0.63 - 11.03 ] | 0 | 0 | 0 | 0 | 0 | 0 |
| Sara tick phlebovirus | 0 | 0 | 0 | 0 | 0 | 0 | 1/15 (1.23)  [ 0.07 - 6.21 ] | 0 | 0 | 0 | 2/9 (9.11)  [ 1.62 - 31.61 ] | 0 | 0 | 0 | 0 | 0 |
| Xue-Cheng virus | 0 | 0 | 0 | 0 | 1/11 (3.62)  [ 0.22 - 16.51 ] | 1/11 (2.65)  [ 0.16 - 12.24 ] | 0 | 0 | 0 | 0 | 0 | 0 | 0 | 0 | 0 | 0 |
| Ji'an nairovirus | 0 | 0 | 0 | 0 | 1/11 (3.41)  [ 0.22 - 15.12 ] | 0 | 1/15 (1.14)  [ 0.07 - 5.35 ] | 0 | 0 | 0 | 0 | 0 | 0 | 0 | 0 | 0 |
| Yanggou tick virus | 0 | 0 | 0 | 0 | 0 | 0 | 0 | 0 | 0 | 0 | 0 | 2/44 (0.54)  [ 0.10 - 1.77 ] | 0 | 0 | 0 | 0 |
| Manly virus | 0 | 0 | 0 | 0 | 0 | 0 | 0 | 0 | 1/17 (0.85)  [ 0.05 - 4.05 ] | 0 | 0 | 1/44 (0.27)  [ 0.02 - 1.30 ] | 0 | 0 | 0 | 0 |
| Jilin partiti-like virus 1 | 0 | 0 | 0 | 0 | 0 | 0 | 1/15 (1.14)  [ 0.07 - 5.40 ] | 0 | 0 | 0 | 1/9 (4.15)  [ 0.22 - 22.15 ] | 0 | 0 | 0 | 0 | 0 |
| Tahe rhabdovirus 1 | 0 | 0 | 0 | 0 | 0 | 0 | 0 | 0 | 0 | 1/12 (1.55)  [ 0.09 - 7.21 ] | 0 | 1/44 (0.27)  [ 0.02 - 1.30 ] | 0 | 0 | 0 | 0 |
| *Ixodes scapularis* associated virus 1 | 0 | 0 | 0 | 0 | 0 | 0 | 1/15 (1.14)  [ 0.07 - 5.40 ] | 0 | 0 | 0 | 0 | 0 | 0 | 0 | 0 | 0 |
| Beiji nairovirus | 0 | 0 | 1/11 (2.19)  [ 0.13 - 10.50 ] | 0 | 0 | 0 | 0 | 0 | 0 | 0 | 0 | 0 | 0 | 0 | 0 | 0 |
| Mukawa phlebovirus | 0 | 0 | 0 | 0 | 0 | 0 | 0 | 0 | 0 | 0 | 0 | 0 | 0 | 0 | 0 | 1/5 (5.00)  [ 0.39 - 21.81 ] |
| Ningxia luteovirus | 0 | 0 | 0 | 0 | 0 | 0 | 0 | 0 | 0 | 0 | 0 | 0 | 0 | 1/7 (2.58)  [ 0.15 - 12.81 ] | 0 | 0 |

The rates at which ticks were infected with viruses were calculated using the bias-corrected MLE method in PooledInfRate software; 95% confidence intervals (CIs) are presented in brackets.
